# Human cytomegalovirus: pathogenesis, prevention, and treatment

**DOI:** 10.1186/s43556-024-00226-7

**Published:** 2024-11-25

**Authors:** Zifang Shang, Xin Li

**Affiliations:** 1 Research Experiment Center, Meizhou Academy of Medical Sciences, Meizhou People’s Hospital, Meizhou, 514031 Guangdong China; 2 Guangdong Engineering Technological Research Center of Clinical Molecular Diagnosis and Antibody Drugs, Meizhou, 514031 Guangdong China; 3 CAS Key Laboratory of Pathogen Microbiology and Immunology, Institute of Microbiology, Chinese Academy of Sciences (CAS), Beijing, 100101 China

**Keywords:** Human cytomegalovirus, Envelope glycoproteins, Immune Evasion, Neutralizing antibodies, Vaccines, Antiviral therapy

## Abstract

Human cytomegalovirus (HCMV) infection remains a significant global health challenge, particularly for immunocompromised individuals and newborns. This comprehensive review synthesizes current knowledge on HCMV pathogenesis, prevention, and treatment strategies. We examine the molecular mechanisms of HCMV entry, focusing on the structure and function of key envelope glycoproteins (gB, gH/gL/gO, gH/gL/pUL128-131) and their interactions with cellular receptors such as PDGFRα, NRP2, and THBD. The review explores HCMV’s sophisticated immune evasion strategies, including interference with pattern recognition receptor signaling, modulation of antigen presentation, and regulation of NK and T cell responses. We highlight recent advancements in developing neutralizing antibodies, various vaccine strategies (live-attenuated, subunit, vector-based, DNA, and mRNA), antiviral compounds (both virus-targeted and host-targeted), and emerging cellular therapies such as TCR-T cell approaches. By integrating insights from structural biology, immunology, and clinical research, we identify critical knowledge gaps and propose future research directions. This analysis aims to stimulate cross-disciplinary collaborations and accelerate the development of more effective prevention and treatment strategies for HCMV infections, addressing a significant unmet medical need.

**Supplementary Information:**

The online version contains supplementary material available at 10.1186/s43556-024-00226-7.

## Introduction

 HCMV infection presents a significant global health challenge, with seroprevalence rates ranging from 70 to 90% in adult populations worldwide. While the infection remains largely asymptomatic in immunocompetent individuals, it can cause severe complications in immunocompromised patients, particularly transplant recipients and HIV/AIDS patients [1]. The virus’s ability to establish lifelong latency with periodic reactivation contributes to its medical significance. Most concerningly, HCMV is the leading infectious cause of congenital birth defects in developed countries, affecting 0.3–2.3% of newborns worldwide. Congenital HCMV infection can result in severe developmental abnormalities, including hearing loss, vision impairment, and neurological disorders [2–6]. Despite over half a century of research efforts, there remains no licensed vaccine, and current therapeutic options are limited by toxicity and the emergence of drug resistance.

As a member of the Betaherpesvirinae subfamily, HCMV possesses the largest genome among known human DNA viruses, spanning approximately 235–250 kb and encoding over 170 proteins and featuring a complex virion structure consisting of a nucleocapsid, tegument layer, and glycoprotein-studded envelope [7]. This genetic complexity underlies the virus’s sophisticated mechanisms for host cell entry, immune evasion, and persistence, which have been gradually unveiled through decades of research. Recent years have witnessed remarkable advances in our understanding of HCMV biology and pathogenesis, driven by technological breakthroughs in structural biology, immunology, and molecular virology. High-resolution structural studies have revealed the intricate architecture of viral glycoprotein complexes and their interactions with host cell receptors [8–12]. Meanwhile, advances in immunology have illuminated the sophisticated mechanisms by which HCMV evades host immune responses. These insights have opened new avenues for therapeutic intervention, including the development of novel antiviral compounds, vaccines, and immunotherapeutic approaches [13]. However, the practical application of these advances has been challenging, highlighting the need for a comprehensive review and analysis of current knowledge to guide future research and therapeutic development.

This review aims to provide an integrated analysis of HCMV pathogenesis, prevention, and treatment strategies. We begin by examining the molecular mechanisms of HCMV entry, which provides the foundation for understanding viral pathogenesis. Based on this mechanistic understanding, we then explore HCMV’s sophisticated immune evasion strategies targeting both innate and adaptive immune responses through multiple coordinated mechanisms. These insights into viral-host interactions inform our subsequent evaluation of therapeutic development, including neutralizing antibodies, various vaccine approaches (live-attenuated, subunit, vector-based, DNA, and mRNA), and both virus-targeted and host-targeted antiviral compounds. Finally, we discuss emerging cellular therapies such as TCR-T cell approaches, which show promise in treating HCMV infections and associated conditions.

By integrating insights from structural biology, immunology, and clinical research, this review seeks to stimulate cross-disciplinary thinking and collaboration. We identify critical areas where further research is needed and propose future directions for developing more effective prevention and treatment strategies. This comprehensive analysis is particularly timely given the continued absence of an effective vaccine and the limitations of current therapeutic options, highlighting the urgent need for innovative approaches to combat HCMV infection and its associated complications.

## HCMV genome structure and life cycle

The full-length HCMV genome is approximately 235–250 kb, with over 70% of the viral genome considered non-essential for viral growth and mostly related to cell tropism and immune evasion [14]. The HCMV genome exhibits an E-type structure, consisting of two unique regions - unique long (UL) and unique short (US), each flanked by terminal (TRL and TRS) and internal (IRL and IRS) inverted repeats [15]. This complex genome encodes ~ 170 canonical open reading frames, which is one-fifth of the coding capacity of non-canonical open reading frames, contributing to the intricate life cycle of HCMV [16, 17].

The HCMV life cycle is characterized by a temporal cascade of gene expression, categorized into immediate early (IE), early (E), and late (L) genes [18]. Upon entry into the host cell, the viral capsid is transported along microtubules to the nuclear pore, where the viral DNA is released into the nucleus [19]. The tegument protein pp71 plays a crucial role in initiating the lytic cycle by promoting the degradation of the cellular repressor Daxx, thereby derepressing viral IE gene expression [20]. IE genes, such as IE1 and IE2, are expressed within hours of infection and are essential for the subsequent expression of E and L genes [18]. E genes, expressed before viral DNA replication, encode proteins necessary for viral genome replication and regulate host cell functions [18]. L genes, expressed after the onset of viral DNA replication, primarily encode structural proteins required for virion assembly [18].

HCMV DNA replication occurs in distinct nuclear compartments and involves both viral and cellular factors [21]. The viral DNA polymerase (UL54) and its processivity factor (UL44) are key components of the replication machinery [22]. Following genome replication, capsid assembly and DNA packaging occur in the nucleus [23].The nuclear egress of newly formed capsids is mediated by the nuclear egress complex (NEC), composed of viral proteins UL50 and UL53 [24]. This process involves a unique envelopment-deenvelopment mechanism at the nuclear membrane [24]. In the cytoplasm, final tegumentation and secondary envelopment take place in the viral assembly compartment (vAC), a juxtanuclear structure formed by the reorganization of cellular organelles [25].The HCMV replication cycle in fibroblasts typically spans approximately 72–96 h, culminating in the release of infectious virions and destruction of the host cell [26]. However, the duration and efficiency of replication can vary depending on factors such as cell type, viral strain, and multiplicity of infection (MOI) [26, 27].Importantly, HCMV can establish latency in certain cell types, particularly in CD34^+^ hematopoietic progenitor cells and CD14^+^ monocytes [28]. During latency, viral gene expression is highly restricted, with only a subset of viral transcripts, including latency-associated transcripts, being expressed [28]. Reactivation from latency can occur under specific conditions, such as cellular differentiation or immune suppression, leading to productive infection and potential clinical manifestations [28].

## Molecular mechanisms of HCMV entry

The molecular mechanisms of HCMV entry involve complex interactions between viral envelope glycoproteins and host cell receptors. This section examines these interactions in detail, including the role of different glycoprotein complexes, their structural features, and their conservation across viral strains.

### Role of envelope glycoproteins

**Fig. 1:**
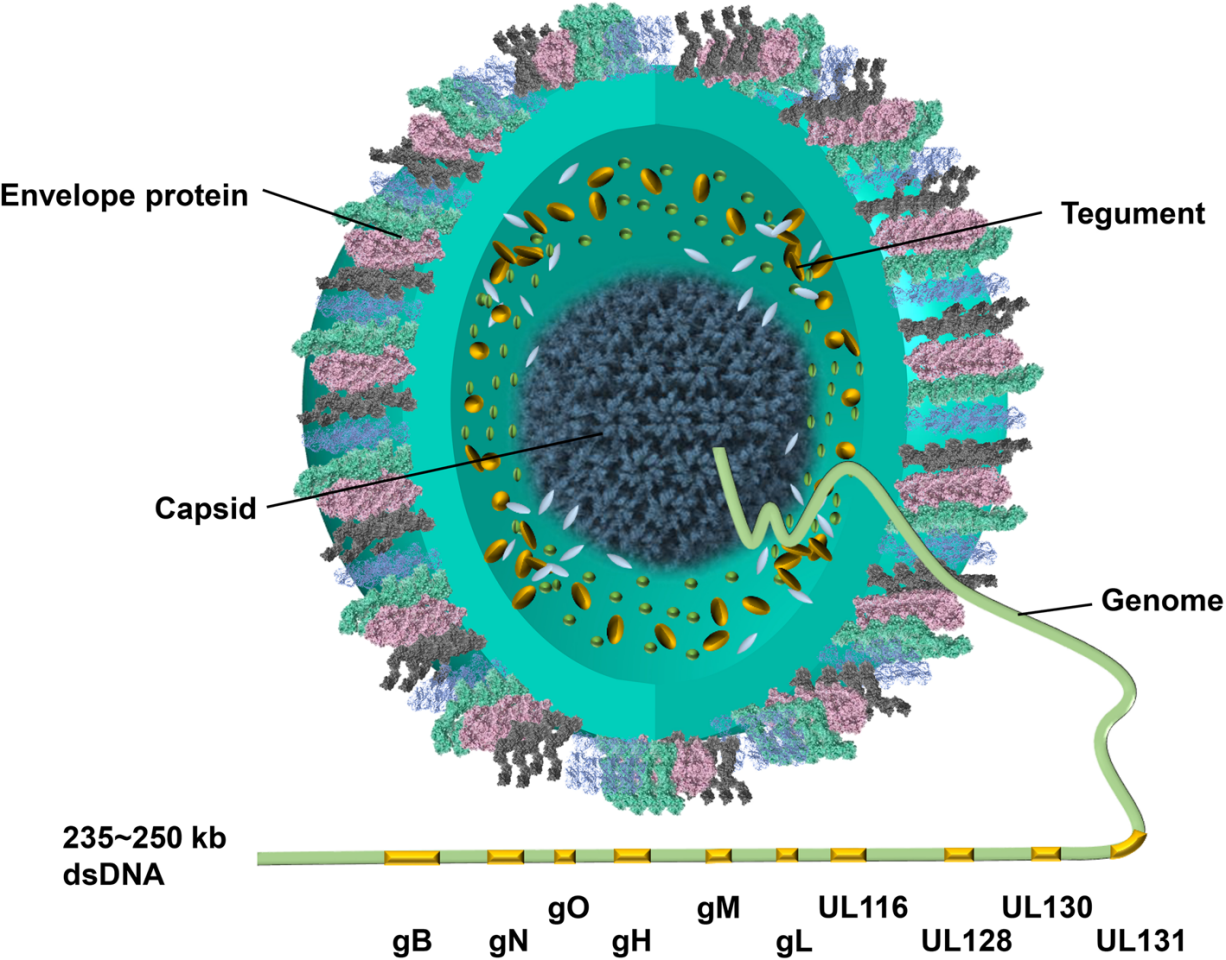
Structure of the human cytomegalovirus (HCMV) virion and its components. The HCMV genome is encapsidated within the capsid, surrounded by the tegument layer, and an outer lipid bilayer envelope embedded with multiple envelope glycoproteins. The genome length is approximately 235–250 kb, encoding envelope glycoproteins such as gB, gM, gN, gH, gL, gO, UL128, UL130, UL131, and UL116

The envelope glycoproteins embedded in the outermost virion envelope play major roles in binding to host cells, viral entry, and in some cases immune evasion [29–33]. Some minor differences in these envelope glycoproteins have been shown to have a dramatic impact on the infectivity of the virus and its ability to induce syncytium formation [34]. Genes encoding these components are located mostly in the UL regions (UL55(gB), UL100(gM), UL73(gN), UL75(gH), UL115(gL), UL116, UL119, UL74(gO) UL128, UL130, UL131(gpUL128, gpUL130, gpUL131), UL132(gp42), UL78, UL1, UL4), some RL segments (RL11(gp34), RL12(gp95), RL13(gpRL13)) and other loci (US27, US28). These envelope glycoproteins commonly form homo-multimers or hetero-multimers such as gM/gN, gB, gH/gL/gO, gH/gL/gpUL128/gpUL130/gpUL131, and the newly identified gH/UL116 (Fig. 1).

### Glycoprotein interactions with host cells

**Fig. 2:**
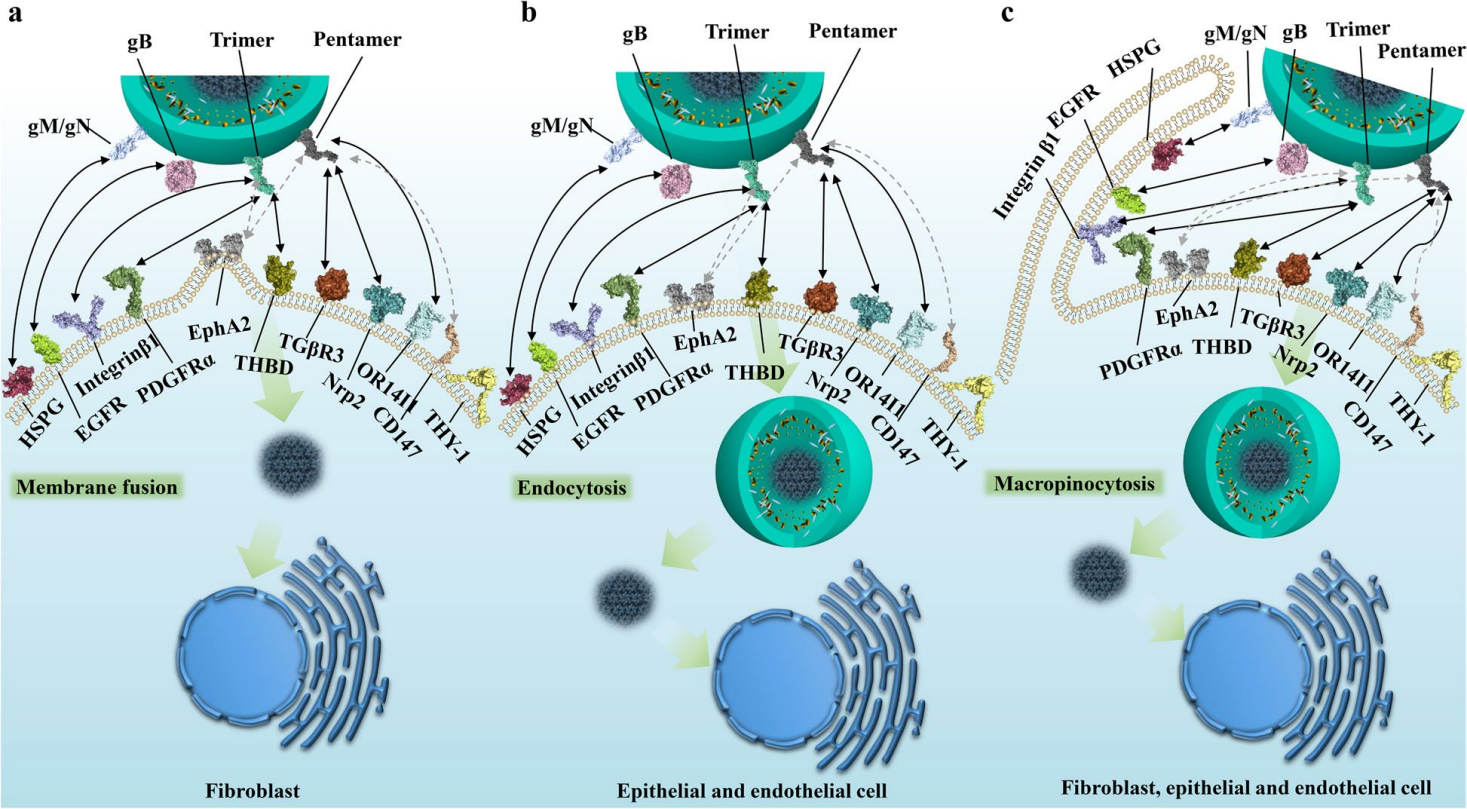
The modes of HCMV cell entry and interactions of envelope glycoproteins with cellular receptors. This figure illustrates the complex interactions between HCMV envelope glycoproteins and cellular receptors, demonstrating different entry pathways in various cell types. In fibroblasts (**a**), entry occurs through membrane fusion at neutral pH, where the gM/gN complex interacts with heparan sulfate proteoglycans (HSPGs), the trimer (gH/gL/gO) binds to PDGFRα and TGFβR3, gB interacts with HSPGs and potentially EGFR, while gH/gL components may interact with integrin β1 and EphA2. CD147 acts as a cofactor promoting entry. In epithelial and endothelial cells (**b**), entry occurs through pH-dependent endocytosis, requiring the pentamer complex (gH/gL/UL128/UL130/UL131) which binds to TGFβR3, Nrp2, or THBD (specifically on endothelial cells). OR14I1 serves as an additional pentamer-dependent receptor on epithelial cells, while CD46 may be involved in downstream entry steps. A macropinocytosis-like entry process (**c**), applicable to fibroblasts, epithelial, and endothelial cells, is activated by integrin and PDGFRα signaling, with THY-1 acting as a cofactor. This mechanism allows HCMV to transport its dsDNA genome into the nucleus. Dashed arrows in the figure indicate potential interactions between viral glycoproteins and cellular receptors that may contribute to the entry process but are not definitively established

The gM/gN complex is considered the most abundant on the HCMV virion surface [35] and initially interacts with cell surface heparan sulfate proteoglycans [36] (Fig. 2), potentially increasing HCMV virion concentration on the cell surface and promoting further interactions crucial for attachment during viral spread, particularly initiating infection in fibroblasts [37]. Magdalena et al. found that an acidic cluster and tyrosine-based sorting motif in the cytoplasmic tail of gM are essential for viral replication and may interact with host proteins regulating gM/gN trafficking rates [38].

gB is considered to be involved in membrane fusion and host cell entry, and it also interacts with heparan sulfate proteoglycans (HSPG) [39]. Additionally, the epidermal growth factor receptor (EGFR) is thought to mediate HCMV infection by interacting with gB and activating downstream signaling pathways such as PI3K [40]. For mononuclear cells, activation of EGFR/PI3K induces cell motility and transendothelial migration, which may facilitate the spread of latently infected cells [41, 42]. Similarly, in fibroblasts, gB has been shown through co-immunoprecipitation experiments to directly interact with PDGFRα, leading to tyrosine phosphorylation of the PDGFRα receptor, subsequent binding to the p85 regulatory subunit of PI3K, and induction of Akt phosphorylation [43]. However, the recent high-resolution structure of the gH/gL/gO-PDGFRα complex has provided different insights into HCMV receptor recognition [11].

**Fig. 3:**
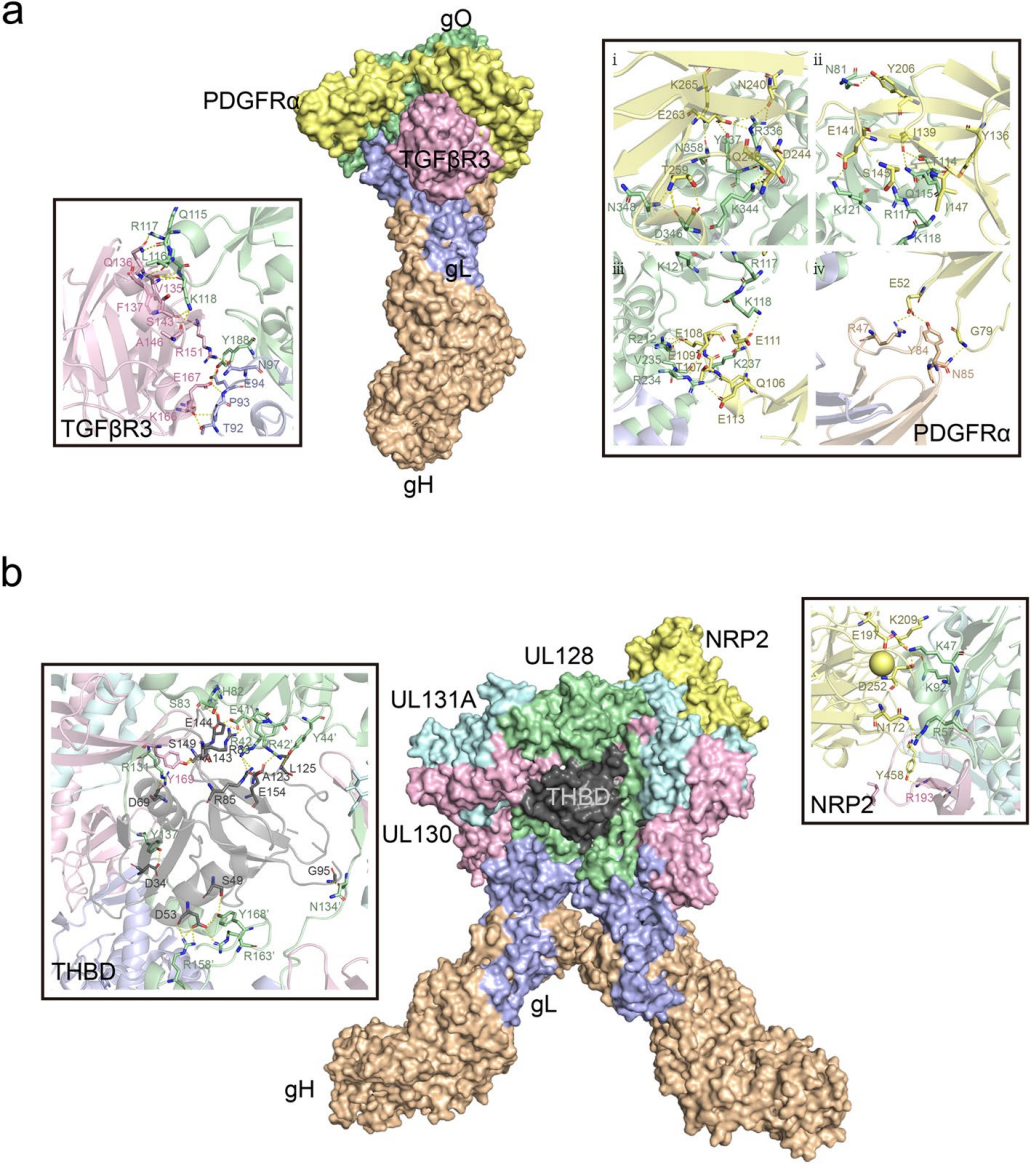
Schematic illustration of the trimer binding to PDGFRα and TGFBR3, and the pentamer binding to Nrp2 and THBD. (**a**) Front view of the overall region where the HCMV trimer binds to PDGFRα (light yellow) and TGFβR3 (pink), with close-up views on either side showing the interaction regions of gO with PDGFRα and TGFβR3, highlighting the key interacting residue sites. (**b**) Front view of the overall region where the HCMV pentamer binds to NRP2 (light yellow) and THMB (dark gray), with close-up views on either side showing the interaction regions of ULs with NRP2 and THMB, highlighting the key interacting residue sites

In the heterotrimeric gH/gL/gO and heteropentameric gH/gL/gpUL128/gpUL130/gpUL131 complexes, with gH/gL as the common component, gH directly binds integrin β1 [44], altering normal intracellular Akt signaling [45], and together with the stimulatory signals from gB, sustains viral survival and persistence. Zhu et al. proposed that the extracellular domain I of gB may contact the gH subunit of gH/gL. Receptor binding by gH/gL triggers conformational changes in the gB endodomains, thereby initiating virus-cell membrane fusion [46]. Recently, Dong et al. discovered that EphA2 on the surface of human glioblastoma cells is a key cellular factor mediating HCMV infection through binding to the gH/gL complex and facilitating membrane fusion [47]. The head regions of these two heteromultimeric complexes are involved in recognition and binding to different host cell types. For instance, some identified receptors such as Nrp2 and the TGFβR3 receptor are expressed in epithelial, endothelial, and fibroblast cells [48]. PDGFRα is highly expressed in fibroblasts and some epithelial cells but has relatively low expression in endothelial cells [49–52], while THBD is primarily enriched in endothelial cells [53], with some expression in fibroblasts and epithelial cells [54–56] PDGFRα is the receptor mediating HCMV entry into fibroblasts [48, 57], with the trimeric virion surface complex primarily binding to PDGFRα through the gO head region with nanomolar affinity [11] (Fig. 3a). Additionally, TGFβR3, which is expressed in epithelial, endothelial, and fibroblast cells, has also been identified as a potential high-affinity binding partner for the trimer [48], specifically binding to the gO head region of the trimer. However, it competes with PDGFRα on the fibroblast surface for binding, acting as an independent receptor, which may be related to the involvement of a broader range of cell types in infection [11]. Nrp2 is the receptor for the HCMV pentamer on epithelial, endothelial, and neuronal cells [58, 59], while THBD is the endothelial cell-specific receptor for the HCMV pentamer, with both receptors exhibiting nanomolar affinities in vitro [48]. High-resolution three-dimensional structures reveal that the Nrp2-pentamer and THBD-pentamer complexes share an overlapping binding site [9, 10]. Similar to the trimeric binding mode, NRP2 and THBD both bind to the UL protein head region of the pentamer, with the common interaction site located at the interface between UL128 and gL. Nrp2 primarily binds to the UL128 and UL131A regions, while THBD mainly binds to the UL128 and UL130 subunits (Fig. 3b). Concurrently, CD46 is considered a factor involved in the downstream entry steps of virus binding to the epithelial cell surface [60]. Furthermore, OR14I1, a multi-pass membrane protein, has also been identified as an additional pentamer-dependent host receptor on the epithelial cell surface [61], which is required for HCMV attachment, epithelial cell infection, and AKT signaling activation. Moreover, proteins located on the surfaces of various cell types have been reported to play important roles in promoting CMV infection. CD147 facilitates the entry of pentamer-expressing HCMV into epithelial and endothelial cells, subsequently inducing cell-cell fusion [62]; however, this molecule has been reported to have similar functions in other viruses, including SARS-CoV-2 [63, 64], HIV [65], and Measles Virus [66].

gH/UL116 is considered a new gH-based complex. UL116 has been shown to compete with gL for binding to gH [67]. Further studies have revealed that UL116 promotes the expression of the gH/gL complex, which is essential for the production of infectious virions, but is influenced by a viral endoplasmic reticulum-resident glycoprotein, UL148 [68]. Some other membrane proteins are involved in immune modulation rather than cell attachment and entry. Proteins such as RL11 (gp34) [69], RL12 (gp95) [70], RL13 (gpRL13) [70], and UL119 (gp68) [71] can act as viral IgG FcγRs, binding and internalizing human immunoglobulins on the surface of infected cells.

### Virus-host cell membrane fusion

After virus particles bind to receptors on host cells via attachment and receptor binding, CMV primarily enters through pH-dependent and pH-independent endocytic pathways, with the specific entry mode depending on the susceptible cell type. HCMV entry into fibroblasts occurs through a pH-independent mechanism involving plasma membrane fusion, mediated by the envelope glycoprotein complexes comprising gB, gH/gL, and/or gH/gL/gO. In contrast, HCMV entry into epithelial, endothelial, and neuronal cells occurs through a pH-dependent fusion mechanism mediated by endocytosis, which requires an additional pentamer glycoprotein complex [14]. Additionally, HCMV exhibits a macropinocytosis-like pathway independent of clathrin, and the activation of this pathway depends on integrin and PDGFR signaling [72]. Furthermore, in specific cell types, a clathrin-independent endocytic vesicle trafficking protein, THY-1, is thought to effectively promote this macropinocytosis-like behavior [73, 74].

### Sequence conservation and structural analysis of key envelope glycoproteins

#### gM/gN

gM is a type III membrane protein containing 7 potential transmembrane domains, with a molecular weight of approximately 42–45 kDa. The amino acid sequence exhibits an average 99% homology across different strains [29]. gN is a heavily glycosylated type I membrane protein with a theoretical molecular weight of 15–18 kDa and 81% sequence conservation. gN is extensively glycosylated, containing both N-linked and O-linked glycosylations, with the latter being more abundant, resulting in an apparent molecular weight of 39–53 kDa. The gM/gN binary complex is formed via a disulfide bond between cysteine 44 of gM and cysteine 90 of gN [75].

#### gB

**Fig. 4:**
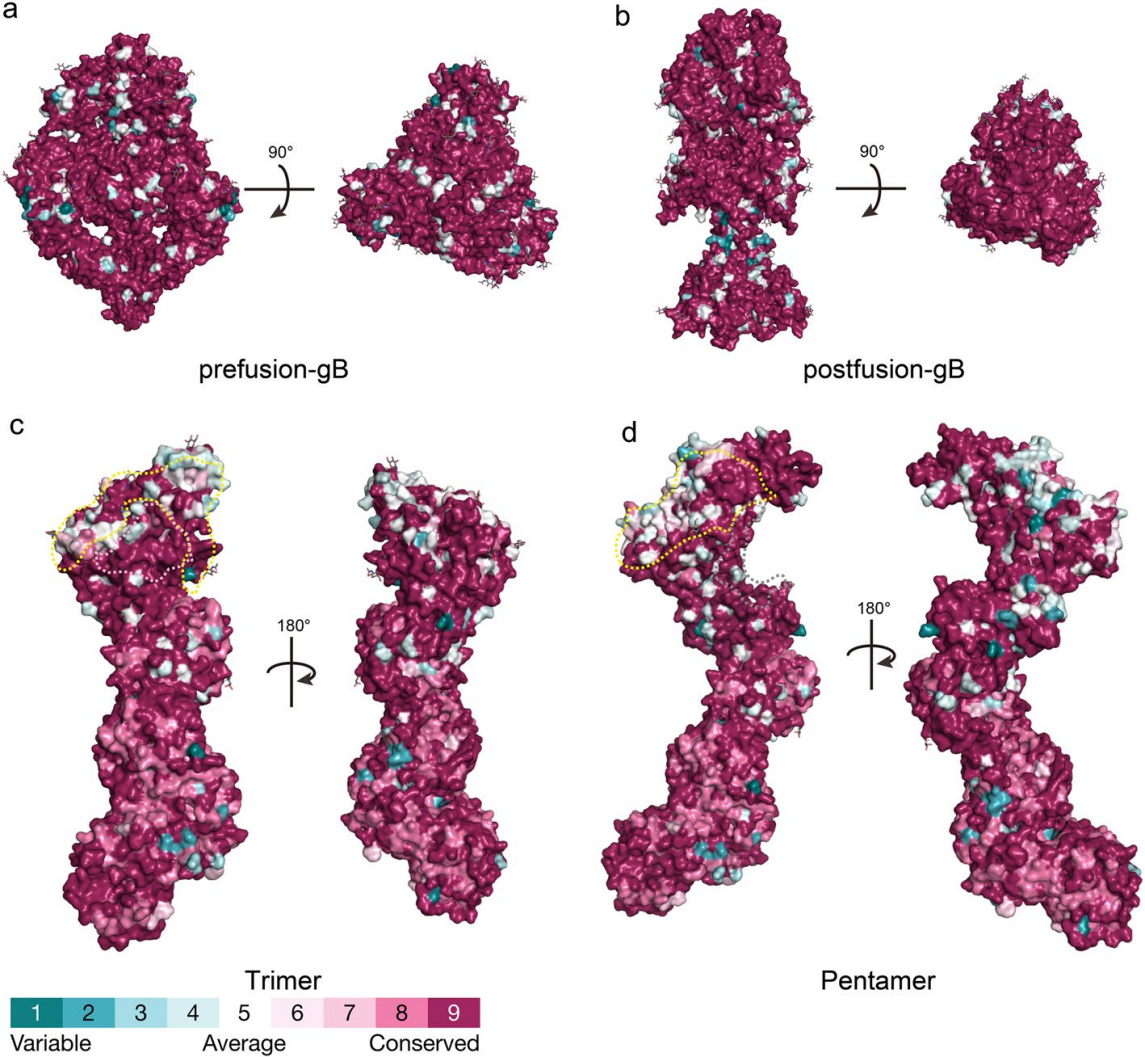
Conservation analysis of the overall structures of HCMV pre-fusion gB, post-fusion gB, trimer, and pentamer. Panels **a**-**b** represent the three-dimensional conservation analysis of 197 gB protein sequences, with the former showing the pre-fusion gB conformation and the latter showing the post-fusion gB conformation. Panel **c** shows the three-dimensional conservation analysis of the trimeric conformation based on 85 gH, 101 gL, and 127 gO sequences. The light-yellow dashed area and pink area represent the interaction regions of gO with PDGFRα and TGFβR3, respectively. Panel **d** shows the three-dimensional conservation analysis of the pentameric conformation based on 85 gH, 101 gL, 73 UL128, 147 UL130, and 41 UL131 sequences. The light-yellow dashed area and dark gray dashed area represent the interaction regions of ULs with NRP2 and THMB, respectively. The Stick representations on each overall structure surface indicate glycosylation sites. The conservation analysis was performed using the ConSurf server and visualized on the protein structures

gB is a type III membrane protein of approximately 900 amino acids. The gB precursor (150 kDa) is cleaved by the furin protease at codon 460 into gp116 and gp55 subunits, which remain covalently linked by disulfide bonds [76]. gB exists in two conformations: a metastable pre-fusion form and a highly stable post-fusion form (Fig. 4a-b). It is currently believed that receptor binding to the trimer and pentamer complexes triggers an irreversible transition of gB from the pre-fusion to post-fusion conformation, resulting in a dramatic rearrangement of the ectodomain during membrane fusion. The pre-fusion gB, which has a cauliflower-like shape with a diameter of ~ 100 Å and a height of ~ 110 Å, transforms into a narrower and taller celery-like shape with a diameter of ~ 70 Å and a height of ~ 170 Å [12]. Approximately 55% of the molecules exhibit the post-fusion conformation of gB, while 13% display the pre-fusion conformation. Overall, the relative positions and orientations of domains I, II, III, and IV in the pre-fusion gB differ significantly from the post-fusion conformation, although the internal folding of each domain remains largely unchanged. During fusion, domains I and II rotate as a rigid body by ~ 180° relative to domains III and IV. In the pre-fusion gB, the domain V is partially buried within the molecule, but during fusion, it undergoes a major refolding and extrusion, forming new interactions with the helices of domain III and domains I/II. Furthermore, the pre-fusion gB contains a membrane-proximal region composed of two amphipathic helices proposed to be involved in membrane fusion, but this region cannot be modeled in the post-fusion structure.

The gB amino acid sequence exhibits an average conservation of 96.81% (Fig. 4a-b), although multiple genotypes may be observed from different body sites within the same patient [77]. Additionally, gB has 18 potential N-linked glycosylation sites and 2 O-linked glycosylation sites [78].

#### gH/gL complex

The trimer adopts a boot-like structure with a length of ~ 170 Å and a width of ~ 70 Å, with 19 N-linked glycosylation sites distributed asymmetrically along the gH/gL/gO complex: 5 on gH, 1 on gL, and 13 on gO [11]. The pentamer has a spiral shape with a length of 180 Å and a width of 30–80 Å, and 10 glycosylation sites on its surface: 6 on gH, 1 on gL, and 3 on the UL proteins [79]. The trimer and pentamer share the gH and gL subunits but differ in their distal subunit composition, comprising gO and UL128/UL130/UL131A, respectively. gH is a type I transmembrane protein of approximately 742 amino acids with a molecular weight of ~ 85 kDa. Its structure is divided into four domains (DI-DIV), extending from the membrane-proximal C-terminus to the N-terminal region (DI), where DI folds together with the membrane-distal region of gL. The gH protein sequence is highly conserved, with 96.10% homology. Variable regions are primarily due to codons encoding the signal peptide [29]. The gL protein is 278 amino acids long, with a molecular weight of ~ 30 kDa, and its amino acid sequence exhibits high conservation across different strains, with 97.48% homology. Cysteine residues at positions 47 and 54 of gL form disulfide bonds with cysteine residues at positions 95 and 59 of gH, respectively, stabilizing the gH/gL interaction [79]. The distal end of the gL subunit interacts with gO in the trimer and with the UL128 and UL130 subunits in the pentamer, specifically forming disulfide bonds between gL C144 and gO C343, and between gL C144 and UL128 C162.

#### gH/gL head region: gO

gO consists of 457–472 amino acids and exhibits relatively low sequence conservation (75.9% homology) among HCMV envelope proteins. However, when mapped onto the three-dimensional structure, large highly conserved surface areas are observed in the N-terminal and C-terminal domains of the gO protein (Fig. 4c). TGFβRIII and PDGFRα bind to these conserved regions (Fig. 4c), with the area where TGFβRIII binds being particularly conserved. These regions are thought to overlap with positively charged areas [11]. Moreover, the glycosylation sites on the gO subunit are not uniformly distributed but are clustered on one side of the trimeric protein, leaving the other side non-glycosylated.

#### gH/gL head region: UL128/UL130/UL131A

UL128, UL130, and UL131A consist of approximately 117, 214, and 129 amino acids, respectively, with sequence homologies greater than 98%, exhibiting high conservation (Fig. 4d). The receptors NRP2 and THBD bind to highly conserved amino acid residues in this region, with the area where THBD binds being particularly conserved (Fig. 4d). Overall, the ULs fold into a core composed of α/β domains, with chemokine-like domains exposed on the side wings [9, 79]. The interaction interfaces between UL128, UL130, and UL131A are relatively small. Specifically, the C-termini of UL131A and UL130 form a core α/β structure, and some of their arginine pairs may become protonated at acidic pH, potentially related to the pH-dependent endocytic entry pathway. The N-terminus of UL130 forms a chemokine-like domain that interacts with the N-terminal extension of gL, while the N-terminus of UL128 also forms a chemokine-like globular domain connected to the UL130-UL131A α/β core [9, 79]. Additionally, UL128 is connected to the gH/gL complex via a 50 Å flexible linker, with its C-terminal short α-helix (UL128-α3) inserted into the N-terminal three-helix bundle of gL. Overall, UL128, UL130, and UL131A assemble into a flexible complex connected to gH/gL through multiple interfaces, which may be related to the process of HCMV entry into host cells.

#### gH/UL116 complex

Additionally, UL116 is thought to directly complex with gH [67]. It consists of 313–315 amino acids with a predicted molecular weight of 34 kDa and has 14 predicted N-linked glycosylation sites on its surface. The UL116 protein sequence is estimated to have 94.92% conservation across different HCMV strains (based on the alignment of 215 sequences). According to AlfaFold2 structure prediction, UL116 is relatively short, structurally loose protein rich in disordered coils and charged residue clusters.

## HCMV immune evasion strategies

HCMV has evolved sophisticated mechanisms to evade host immune responses, targeting both innate and adaptive immunity. This section explores these immune evasion strategies, their molecular basis, and their implications for viral persistence and pathogenesis.

### Interference with innate immune pathways

**Fig. 5:**
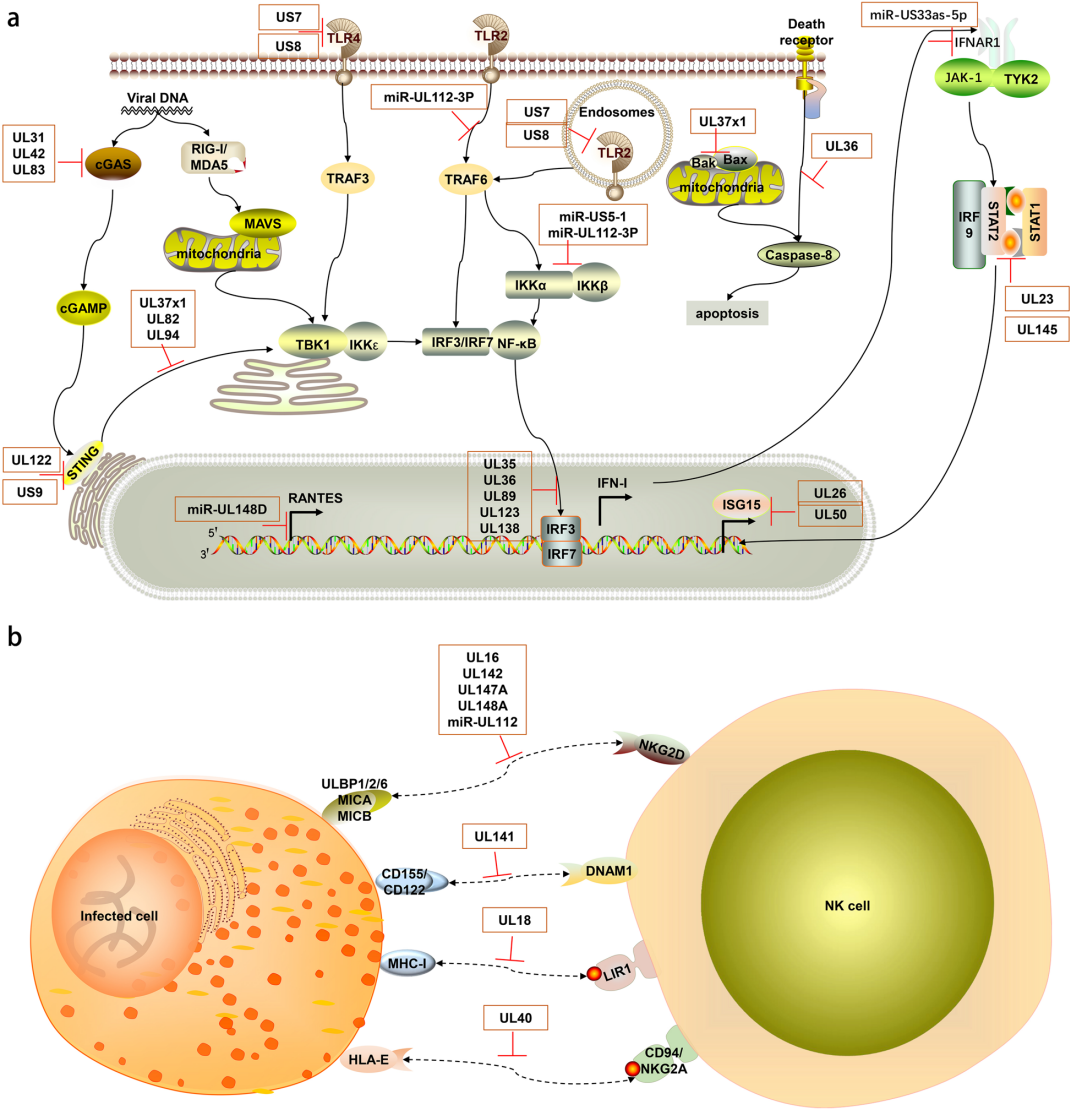
Schematic diagram of the mechanisms by which HCMV evades host innate immune pathways. The host innate immunity and immune evasion mediated by HCMV. TLRs, located at both the plasma membrane and endosomes, sense different viral components. The cGAS-STING pathway detects viral DNA. These pathways activate IRF3/7 or NF-κB, inducing IFN-I and inflammatory cytokines. IFN-I stimulates ISG expression via the JAK-STAT pathway to limit HCMV. The IFN-I, inflammatory cytokines, and ISGs are induced for antiviral immunity, but HCMV proteins and miRNAs highlighted in the orange boxes can hijack multiple steps of these signaling pathways, effectively suppressing these immune reactions. In part (**a**), UL31, UL42, and UL83 (pp65) inhibit cGAS, while UL37 × 1, UL82, and UL94 block STING trafficking. US7 and US8 suppress TLR3 and TLR4 signaling, and miR-UL112-3P targets TLR2. UL122 (IE86) induces STING degradation, and US9 blocks STING-TBK1 interaction. UL35, UL36, UL89 (pp65), and UL138 inhibit IRF3 phosphorylation. miR-US33as-5p targets IFNAR1, UL23 blocks STAT1 nuclear translocation, and UL145 induces STAT2 degradation. miR-US5-1 and miR-UL112-3P downregulate IKKα and IKKβ. UL36 inhibits caspase-8 activation, and UL37 × 1 inhibits Bax and Bak. UL26 and UL50 suppress ISGylation. In part (**b**), HCMV evades NK cell responses. UL16 binds to NKG2D ligands MICB, ULBP1/2/6. UL142, UL147A, UL148A, and miR-UL112 downregulate MICA/B. UL141 downregulates DNAM-1 ligands CD155 and CD112. UL18 mimics MHC-I molecules, binding to LIR-1. UL40 mimics HLA-E ligands, preventing NK cell-mediated lysis. This figure was created using ScienceSlides 2016

Human cytomegalovirus (HCMV) has evolved complex and sophisticated strategies to evade host innate immune responses through its long-term co-evolution with the host. These strategies involve various stages of the viral life cycle, from viral invasion and gene expression to viral assembly and release, reflecting the ongoing battle between HCMV and the host immune system. HCMV primarily suppresses and modulates innate immune responses through the following mechanisms ((Fig. 5a-b).

#### Interference with pattern recognition receptor (PRR) signaling pathways

HCMV encodes multiple proteins and microRNAs to target and inhibit PRR signaling pathways, which is a key strategy for viral evasion of innate immune recognition. For example, viral proteins UL31 and UL42 can bind to the cytoplasmic DNA sensor cGAS, inhibiting its enzymatic activity and DNA binding ability, thereby blocking the cGAS-STING signaling pathway [80, 81]. This inhibition not only affects cGAS recognition of viral DNA but may also interfere with cellular recognition of other DNA pathogens. pp65 (UL83) protein, one of the most abundant tegument proteins of HCMV, can also interact with cGAS, preventing its binding to STING [82]. This multi-targeted strategy against the cGAS-STING pathway demonstrates HCMV’s emphasis on this critical innate immune pathway.

Furthermore, proteins such as UL37 × 1, UL82, and UL94 can block STING trafficking from the endoplasmic reticulum to the Golgi apparatus, suppressing downstream signal transduction [83–85]. This interference with STING trafficking affects not only the cGAS-STING pathway but potentially other antiviral signaling pathways dependent on STING. Notably, HCMV also regulates TLR-mediated innate immune responses by targeting Toll-like receptor 2 (TLR2) through hcmv-miR-UL112-3P [86]. Additionally, HCMV glycoproteins US7 and US8 have been identified as key suppressors of TLR3 and TLR4 signaling [87]. US7 promotes ubiquitin-dependent degradation of these TLRs, while US8 disrupts TLR3-UNC93B1 association and targets TLR4 to lysosomes for degradation [87]. This simultaneous interference with multiple PRR pathways reflects the comprehensiveness and complexity of HCMV’s evasion of innate immune recognition.

#### Suppression of type I interferon (IFN-I) production and signaling

The IFN-I system is a key defense line against viral infections, and HCMV has evolved multiple strategies to suppress IFN-I production and signaling. For instance, IE86 encoded by UL122 can induce STING degradation, while US9 can block the interaction between STING and TBK1 [88–90], both directly interfering with IFN-I production. Additionally, UL123 is also associated with IFN-I downregulation [91]. Proteins such as UL35, UL36, UL138, and pp65 (pUL89) can inhibit IRF3 phosphorylation [92–95], a crucial step in IFN-I gene transcription activation. This multi-level interference ensures HCMV’s effective suppression of IFN-I production. UL138 suppresses IE gene expression to attenuate IFN-I response, contributing to the establishment of latent infection [95]. This dual action of immune evasion and latency promotion demonstrates the virus’s sophisticated manipulation of host cell processes.

Regarding IFN-I signal transduction, HCMV employs multiple strategies as well. hcmv-miR-US33as-5p can target the IFN-α/β receptor subunit 1 (IFNAR1), interfering with IFN signaling [96]. This affects not only the autocrine effects of IFN-I but may also impact the response of nearby uninfected cells to IFN-I. Furthermore, HCMV can inhibit IFN-γ-induced antiviral gene expression by binding to N-myc interacting protein (Nmi) through UL23 protein, blocking STAT1 nuclear translocation [97]. The two protein isoforms (pUL145-Long and pUL145-Short) encoded by the UL145 gene can mimic cellular DDB1-cullin-associated factors (DCAFs), recruiting DDB1-containing ubiquitin ligases to induce proteasomal degradation of STAT2, thereby further suppressing IFN signaling [98]. This dual interference with both type I and II IFN systems greatly enhances HCMV’s immune evasion capabilities.

Moreover, ISGylation inhibits HCMV growth by downregulating viral gene expression and virion release. UL26 has been found to interact with ISG15, UBE1L, and Herc5 to suppress virus-induced ISGylation [99, 100]. UL50 has been discovered to interact with an ER-associated ubiquitin E3 ligase RNF170, regulating ISGylation and inhibiting ISGylation by causing proteasomal degradation of UBE1L [101].

#### Regulation of NK cell activity

Natural killer (NK) cells are key effector cells of the innate immune system against viral infections, and HCMV has evolved various mechanisms to regulate NK cell activity. For example, UL16 can bind to NKG2D ligands MICB, ULBP1/2/6, preventing their interaction with NKG2D [102]. UL142, UL147A, and UL148A downregulate the NKG2D ligand MICA, further weakening the recognition and killing ability of NK cells [103–105]. This strategy not only reduces NK cell activation but may also affect NK cell-mediated memory-like responses. At the transcriptional level, hcmv-miR-UL112 can suppress MICB expression, thereby reducing NK cell cytotoxicity [106]. This strategy of regulating NK cell activity through miRNA demonstrates the refinement and diversity of HCMV’s immune evasion. UL141 can downregulate the expression of CD155 and CD112, ligands of the NK cell activation receptor DNAM-1 [107], further weakening NK cell recognition and killing ability.

Additionally, HCMV encodes the UL18 protein, an MHC-I-like molecule that can bind to the NK cell inhibitory receptor LIR-1, further suppressing NK cell function [40, 108]. This strategy of mimicking host MHC-I molecules reflects the result of long-term co-evolution between HCMV and the host immune system. Similarly, the leader peptide of UL40 contains peptide sequences resembling HLA-E ligands, thereby preventing NK cell-mediated lysis [109].

#### Interference with inflammatory factor and chemokine production

Inflammatory responses and immune cell recruitment are important mechanisms for controlling viral infections, and HCMV interferes with these processes in various ways. For example, hcmv-miR-US5-1 and hcmv-miR-UL112-3P can downregulate IKKα and IKKβ, inhibiting the NF-κB signaling pathway and thus reducing the production of IL-6, CCL5, IL-1β, and TNF-α [110]. This simultaneous suppression of multiple inflammatory factors may lead to significant weakening of local inflammatory responses.

hcmv-miR-UL148D can specifically inhibit the production of the chemokine RANTES, affecting immune cell recruitment [111]. RANTES plays an important role in T cell and monocyte recruitment, and its inhibition may lead to delayed or weakened antiviral immune responses. Furthermore, HCMV-encoded UL146 and UL147 proteins are homologs of CXC chemokines and may regulate immune cell migration by competing with host chemokines [104, 112]. This strategy of mimicking and interfering with host chemokine networks reflects HCMV’s fine-tuning of the host immune system.

#### Regulation of apoptosis and autophagy

Apoptosis and autophagy are important mechanisms for the host to clear virus-infected cells, and HCMV has evolved various strategies to regulate these processes. In terms of anti-apoptosis, HCMV encodes multiple proteins to inhibit cell apoptosis, such as UL36 inhibiting caspase-8 activation, and UL37 × 1 binding and inhibiting Bax and Bak [113–115]. These strategies not only prolong the survival time of infected cells but also provide more replication time for the virus.

Regarding autophagy regulation, HCMV’s strategies are more complex. For instance, US12 can upregulate ULK1 phosphorylation and LC3-II conversion, promoting autophagy flux [116]. This regulation of autophagy may have multiple effects, potentially facilitating viral particle assembly and release on one hand, and altering the intracellular environment to favor viral replication on the other. Additionally, HCMV may influence antigen presentation and cytokine production by regulating autophagy, further modulating immune responses [117, 118].

In conclusion, HCMV has evolved multiple sophisticated mechanisms to evade and suppress host innate immune responses, including interfering with PRR signaling pathways, suppressing IFN-I production and signaling, regulating NK cell activity, inhibiting inflammatory factor production, and modulating cell apoptosis and autophagy. These strategies do not exist in isolation but are coordinated and complementary, forming a complex network. Through these multi-level, multi-targeted immune evasion strategies, HCMV can effectively evade host innate immune surveillance, creating favorable conditions for viral latent infection and continuous transmission.

### Modulation of adaptive immune pathways

**Fig. 6:**
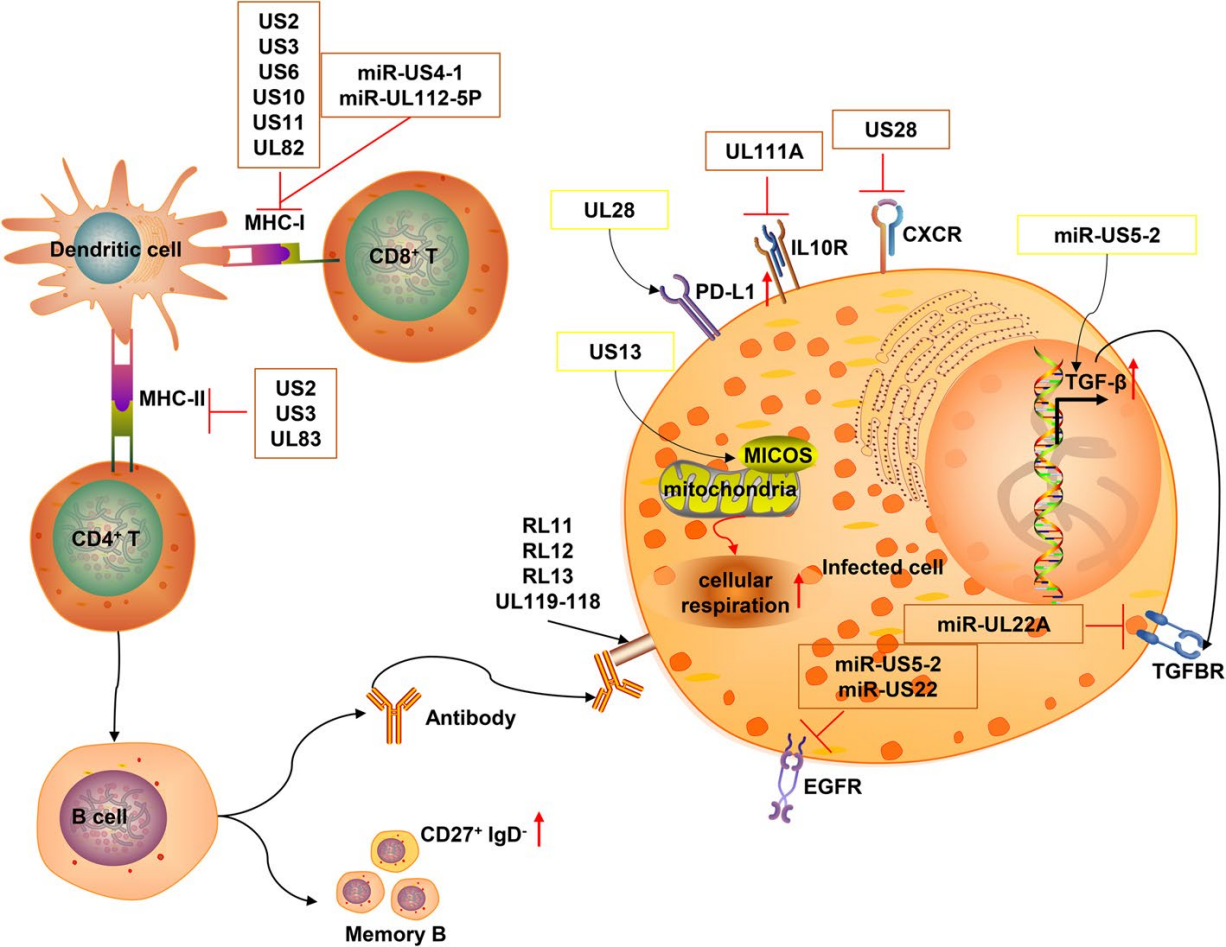
Schematic diagram of the mechanisms by which HCMV evades host adaptive immune pathways. The host adaptive immunity and immune evasion mediated by HCMV. Dendritic cells present antigens via MHC-I and MHC-II to CD8^+^ and CD4^+^ T cells, respectively. B cells produce antibodies and form memory B cells. These processes activate adaptive immune responses against HCMV. However, HCMV proteins and miRNAs highlighted in the orange boxes can hijack multiple steps of these pathways, effectively suppressing these immune reactions. US2, US3, US6, US10, US11, and UL82 interfere with MHC-I expression and transport, while US2, US3, and UL83 (pp65) interfere with MHC-II. miR-US4-1 and miR-UL112-5P target ERAP1, affecting antigen processing. UL111A (cmvIL-10) inhibits T cell function via IL10R, and US28 induces T cell apoptosis through CXCR. UL28 (highlighted in yellow) upregulates PD-L1, inhibiting T cell activity. UL13 (highlighted in yellow) enhances cellular respiration by targeting mitochondrial MICOS complex. RL11, RL12, RL13, and UL119-118 bind to antibody Fc regions, interfering with ADCC. HCMV infection increases CD27^+^ IgD^-^ memory B cells. miR-US5-2 (highlighted in yellow) increases TGF-β production, while miR-UL22A inhibits TGF-β signaling. miR-US5-2 and miR-US22 affect cell proliferation and viral latency by targeting GAB1 and EGR-1, respectively. This figure was created using ScienceSlides 2016

In addition to evading innate immunity, HCMV has evolved multiple strategies to evade host adaptive immune responses. These strategies involve various aspects of T cell and B cell-mediated immune responses, reflecting the long-term co-evolution between HCMV and the host adaptive immune system. HCMV’s strategies to evade adaptive immunity mainly include the following aspects (Fig. 6).

#### Interference with antigen presentation

Antigen presentation is a key step in initiating adaptive immune responses, and HCMV has evolved various mechanisms to interfere with this process. Regarding MHC-I antigen presentation, US2, US11, and UL82 can promote the degradation of MHC-I molecules, while US3, US6, and US10 can prevent MHC-I molecules from being transported to the cell surface [119–121]. These strategies not only reduce the number of MHC-I molecules on the surface of infected cells but also interfere with the normal assembly and transport process of MHC-I molecules.

In terms of antigen processing, hcmv-miR-US4-1 and hcmv-miR-UL112-5P can target the endoplasmic reticulum aminopeptidase ERAP1, affecting the trimming of antigenic peptides [122, 123]. ERAP1 plays a crucial role in trimming antigenic peptides to fit the MHC-I binding groove, and its impaired function may lead to the production of suboptimal antigenic peptides, thereby affecting T cell recognition efficiency. By suppressing IE protein expression (e.g., miR-UL112-1 targeting IE72), HCMV reduces antigen presentation, aiding in maintaining latency [124].

HCMV can also interfere with MHC-II-mediated antigen presentation. US2, US3, and pp65 (UL83) can interfere with the expression and antigen presentation of MHC-II molecules [125–127]. In particular, pp65 (UL83) can alter the subcellular localization of MHC-II molecules, redirecting them to lysosomes for degradation. These mechanisms work together to cause infected cells to evade recognition by both CD8^+^ T cells and CD4^+^ T cells, thereby weakening specific T cell responses.

Furthermore, HCMV may influence antigen presentation by regulating the autophagy process. For example, HCMV may affect antigen processing and loading by regulating the formation and maturation of autophagosomes [118]. This fine-tuning of intracellular membrane dynamics further demonstrates the complexity of HCMV’s immune evasion.

#### Regulation of T cell function

T cells are key effector cells in controlling HCMV infection, and HCMV employs multiple strategies to regulate T cell function. For example, viral IL-10 (cmvIL-10) encoded by UL111A can inhibit T cell proliferation and cytokine production [128]. cmvIL-10 not only acts directly on T cells but may also indirectly affect T cell responses by modulating the function of antigen-presenting cells. US28, as a chemokine receptor homolog, can induce T cell apoptosis [129, 130].

HCMV can also suppress T cell function by inducing PD-L1 expression [131]. The PD-1/PD-L1 pathway is an important immune checkpoint, and its activation can lead to T cell functional exhaustion. For instance, UL23 upregulates the expression and signaling of programmed death ligand 1 (PD-L1), which is responsible for inhibiting multiple aspects of T cell activity, including activation, apoptosis, and IFN-γ secretion, thereby evading immune clearance [131].

Furthermore, HCMV may influence T cell function by regulating T cell metabolism. Recent research has discovered that HCMV’s UL13 protein plays a crucial role in remodeling mitochondrial structure and function [132]. UL13 targets mitochondria and interacts with the MICOS complex, a key regulator of cristae architecture and electron transport chain (ETC) function. By altering mitochondrial cristae structure, UL13 can increase oxidative phosphorylation and cellular respiration, thereby enhancing energy output. This regulation of mitochondrial function not only supports viral replication but may also affect T cell metabolism and function. By manipulating host cell energy metabolism, HCMV may influence T cell activation, proliferation, and effector functions, thus evading T cell-mediated immune responses.

#### Regulation of B cell responses

While T cell-mediated cellular immunity plays a dominant role in controlling Human Cytomegalovirus (HCMV) infection, B cell-mediated humoral immunity should not be overlooked. HCMV interferes with B cell responses through various mechanisms, thereby affecting antibody-mediated immune defense. HCMV infection may lead to the production of autoreactive antibodies, interfering with normal antibody responses. The generation of these autoreactive antibodies may stem from molecular mimicry or immune dysregulation caused by viral infection, further complicating the humoral immune response following HCMV infection. The mechanisms by which HCMV evades antibody-dependent cell-mediated cytotoxicity (ADCC) have been extensively studied. These studies have shown that HCMV encodes multiple glycoproteins capable of binding to the Fc region of IgG, thereby preventing host FcγR activation. Four HCMV glycoproteins have been identified with Fcγ binding capabilities: RL11 (gp34), RL13, RL12 (gp95), and UL119-UL118 (gp68) [70, 133, 134]. These proteins bind to Fcγ in a manner different from host FcγR-Fcγ interactions, potentially avoiding direct competition with host counterparts. For instance, gp68 is thought to bind the interface between the CH2 and CH3 domains of Fc in a 2:1 stoichiometry, while host FcγRΙΙΙ binds to the CH1-CH2 hinge and CH2 domain in a 1:1 binding mode. This strategy may not only affect ADCC but may also interfere with antibody-mediated complement activation [135, 136]. Furthermore, HCMV infection may affect the formation and maintenance of memory B cells. Studies have shown that HCMV infection can lead to changes in memory B cell subpopulation proportions, particularly an increase in CD27^+^ IgD^−^ memory B cells [137]. These changes in memory B cell subsets may affect the quality and durability of long-term antibody responses, creating conditions for HCMV’s long-term persistent infection. Recent studies have emphasized the importance of ADCC in maternal immunity against congenital HCMV (cCMV) transmission. Research has found that higher maternal serum ADCC activation is associated with a lower risk of cCMV transmission, with antibodies against the HCMV immune evasion protein UL16 showing the strongest correlation with ADCC activation [138]. This suggests that ADCC-activating antibodies, especially those targeting UL16, may represent a key protective maternal immune response against cCMV infection. These findings not only deepen our understanding of the interaction between HCMV and the host immune system but also provide important clues for the development of new vaccines and immunotherapy strategies.

#### Regulation of dendritic cell (DC) function

Dendritic cells (DCs) play a crucial role in bridging innate and adaptive immunity during Human Cytomegalovirus (HCMV) infection. HCMV has evolved multiple strategies to modulate DC function, indirectly regulating T and B cell responses. This modulation occurs through various mechanisms. The viral protein cmvIL-10 (UL111A) suppresses DC maturation and antigen presentation capabilities, affecting the expression of co-stimulatory molecules on the DC surface and potentially altering the cytokine profile secreted by DCs, thus influencing T cell polarization [139, 140]. HCMV infection suppresses the production of pro-inflammatory cytokines by DCs, including IL-12 and TNF-α, and impairs their ability to produce cytokines in response to stimuli like LPS or CD40L [141]. Furthermore, HCMV infection has been shown to downregulate CCR7 expression on DCs, impairing their migration to lymph nodes and potentially reducing antigen presentation efficiency [142]. These multifaceted regulations of DC function allow HCMV to intervene in the immune response from its initial stages, targeting DCs to indirectly modulate both innate and adaptive immune responses. This comprehensive strategy contributes significantly to HCMV’s ability to establish persistent infection, highlighting the virus’s sophisticated mechanisms for evading host immunity.

#### Modulation of immune microenvironment

HCMV employs various strategies to modulate the immune microenvironment, facilitating both immune evasion and the establishment of latency. Multiple HCMV proteins and miRNAs regulate the production of cytokines and chemokines, such as inhibiting the secretion of IL-6, TNF-α, and RANTES [143]. Viral proteins UL146 and UL147 may compete with host chemokines to regulate immune cell migration [144]. The virus also manipulates the TGF-β signaling pathway to influence the formation of an immunosuppressive microenvironment. For example, HCMV miR-US5-2 increases TGF-β production by downregulating the transcriptional repressor NGFI-A-binding protein (NAB1), thereby inducing myelosuppression in uninfected CD34^+^ hematopoietic progenitor cells (HPCs), while miR-UL22A inhibits the TGF-β signaling pathway by targeting SMAD3, contributing to the maintenance of the viral genome in a latent state [145]. Recent studies have revealed additional mechanisms: miR-US5-2 targets the GAB1 protein, affecting the EGF signaling pathway and inhibiting cell proliferation, which helps the virus exit the latent phase [146]. Another miRNA, miR-US22, targets the early growth response gene 1 (EGR-1) and exerts the same function [147]. Furthermore, HCMV may recruit histone deacetylases (HDACs) to suppress IE gene expression, regulating epigenetic modifications and thereby maintaining latency [148]. These diverse mechanisms collectively create a microenvironment conducive to viral persistence and latency, while simultaneously hampering effective immune responses.

## Advances in HCMV prevention and treatment

The current preclinical and clinical development pathways for intervening with HCMV surface membrane proteins are primarily focused on monoclonal antibodies, vaccines. In this section, we will summarize some of the impressive achievements made in these areas in recent years.

### Neutralizing antibodies

Monoclonal antibodies, with their advantages of rapid onset, high specificity, and few long-term adverse effects, can serve as potential drugs for treating and preventing viral infections [149]. Over the past decade or so, tremendous progress in monoclonal antibody screening techniques has led to the isolation and identification of many human-derived antibodies specifically targeting different HCMV envelope proteins. Furthermore, advances in antibody engineering and structural biology have enabled the optimization of highly neutralizing antibodies by enhancing their effector functions, prolonging half-lives, and focusing on conserved antibody regions. Currently, several monoclonal antibodies targeting HCMV have undergone multiple clinical trials, but no commercialized antibody-based therapy for clinical use is available (Table 1).

The gM/gN-specific antibody titer is considered unrelated to the neutralizing titer in HCMV-positive donor sera [150]. Currently, the main antibody targets against HCMV are focused on the three envelope protein complexes: gB, gH/gL/gO, and gH/gL/gpUL128/gpUL130/gpUL131.

The gB protein exists in a metastable prefusion conformation and a stable postfusion conformation [12]. The instability of the prefusion gB conformation has hindered its use as a vaccine antigen target, and thus, the existing gB antibodies have been discovered based on the stable postfusion gB. The postfusion gB has five major antigenic domains (ADs), denoted as domains I-V. It has been reported that over 90% and 50% of HCMV-positive donor sera bind to the AD4 and AD5 epitopes of gB, respectively [151]. Spindler et al. determined the structural basis for the AD4 epitope-specific monoclonal antibody SM5-1 [152] and the AD5 epitope-specific monoclonal antibody 1G2 [76] recognizing gB. AD2 is a linear epitope region at the N-terminus of gB, containing the variable II epitope and the highly conserved I epitope. Several AD2 epitope-specific neutralizing antibodies, including 3–25, TRL345, and ITC88, have also been characterized in detail [153, 154]. Studies have shown that humanized neutralizing antibodies specific for the gB AD2 epitope from different donors rely on a set of highly conserved variable region genes (IGHV3-30 and IGKV3-11) for their function, and their antiviral neutralizing activity requires the concerted action of the two Fab portions of the antibody [155]. Recently, Sponholtz et al. produced a prefusion-like conformation of gB, termed gB-C7, using structure-based engineering approaches. However, mouse immunization data suggested that the prefusion-stabilized HCMV gB protein did not offer an advantage over postfusion gB in eliciting potent neutralizing antibodies [156].

As a common component of the trimer and pentamer, gHgL has also been extensively studied for its epitopes, with 11B12 and 13H11 primarily binding to binding domain A of gHgL [157], while MSL09, 3G16, and MCMV5322A bind to the opposite binding domain B of gHgL [158]. Additionally, Ai et al. isolated three highly neutralizing antibodies, PC0012, PC0014, and PC0035, that bind to the gHgL epitope [159]. Recently, Zehner and colleagues isolated 69 neutralizing antibodies that bind to previously unreported regions on gHgL [160].

UL128-131a binds to the CD147 and Neuropilin2 receptors on epithelial and endothelial cells [62], promoting virus particle entry into cells through a pH-dependent endocytic pathway [9, 10]. Many antibodies targeting different components of UL128-131a have also been characterized [161, 162]. Chandramouli et al. revealed the assembly of the pentamer and the molecular structure recognized by two potent neutralizing antibodies, 9I6 and 8I21, with 9I6 primarily binding to the UL128 protein and 8I21 primarily binding to the UL130 protein [79]. The recently resolved cryo-EM structures of the pentamer bound to the NRP2 receptor and four antibodies (1–32, 1-103, 2–18, and 2–25) unveiled the molecular basis for the pentamer’s interaction with NRP-2 and revealed the unique mechanism by which pentamer-specific antibodies neutralize HCMV [9]. Some pentamer-specific neutralizing antibodies, such as 10F7, 10P3, 2C12, 7I13, 1-103, and PC0034 [9, 79, 159], can neutralize HCMV by inhibiting the binding of the pentamer to NRP2. Other potent pentamer-specific neutralizing antibodies, such as 8I21, 2–18, and 2–25, although not blocking the pentamer’s binding to NRP-2, may neutralize HCMV by inhibiting binding to other unknown receptors or preventing conformational changes in the pentamer.

gO is involved in the major binding site for the receptors PDGFRα and TGFβR3. In recent years, Gerna et al. utilized EBV-immortalized B cells to screen for the gO epitope-specific antibody CVB234, which exhibited an in vitro neutralizing IC50 value of only 1–3 µg/mL [157]. This monoclonal antibody has been reported to effectively inhibit the infection of fibroblasts by the AD169 strain and reduce the infection of the epithelial cell line ARPE-19 by the VR1814 strain [57]. Recently, Zehner et al. isolated a new gO-neutralizing monoclonal antibody, CS2it1p2_F7K, and structural analysis revealed that its CDRH3 region interacts with amino acid residues 240–242 of the gO protein [160]. The neutralizing epitope of CS2it1p2_F7K partially overlaps with the PDGFRα binding site, and it neutralizes HCMV infection by inhibiting the binding of PDGFRα to gO, without competing with the TGFβR3 receptor.

Over the past decade, clinical trials have been conducted based on neutralizing antibodies against gB and the pentamer. However, the gB-targeting TCN-202 was terminated in a phase I clinical trial due to adverse events (NCT01594437), while another gB-targeting NPC-21 was used in a phase II clinical study for high-risk kidney transplant recipients with cytomegalovirus infection (NCT04225923). The gH-targeting MSL109 failed to demonstrate efficacy in preventing HCMV infection in clinical trials for the treatment of CMV retinitis [163] (NCT00000836, NCT00001061). Similarly, some antibody cocktail drugs, such as CSJ148 and RG7667 (a mixture of gB and pentamer antibodies, and a mixture of gH and pentamer antibodies, respectively), did not achieve the expected results (NCT02268526, NCT01753167).

**Table 1:** Representative neutralizing antibodies (nAbs) targeting human herpes virus entry complex

Prototypic antibodies	Target site	Method of generation	Reference
SM5-1	gB AD4	B cell immortalization	[151]
1G2	gB AD5	B cell immortalization	[151]
3–25	gB AD2	Single B cell cloning	[164]
TRL345、	gB AD2	CellSpot™ platform	[165]
ITC88	gB AD2	B cell immortalization	[166]
11B12、13H11	gHgL domain A	B cell immortalization	[162]
3G16	gHgL domain B	B cell immortalization	[162]
PC0012、PC0014、PC0035	gHgL	Single B cell cloning	[159]
CS4tt1p1_E3K	gHgL	Single B cell cloning	[160]
9I6、8I21	UL128-UL130-UL131A	B cell immunization	[162]
10F7、10P3、2C12、7I13、1-103	UL128-UL130-UL131A	B cell immunization	[162]
PC0034	UL128-UL130-UL131A	Single B cell cloning	[159]
2–18、2–25	UL128-UL130-UL131A	Single B cell cloning	[164]
CVB234	gO	B cell immunization	[157]
CS2it1p2_F7K	gO	Single B cell cloning	[160]
**Clinical antibody**	**Target site**	**Clinical status**	**Clinical trial number**
TCN-202	gB	Phase l (terminated)	NCT01594437
NPC-21	gB	Phase ll	NCT04225923
MSL109	gH	Phase ll/lll	NCT00000836、NCT00001061
CSJ148	gB and pentamer	Phase ll	NCT02268526
RG7667	gH and pentamer	Phase lla	NCT01753167

### Vaccine development progress

Vaccines are one of the most effective medical interventions for preventing and treating a wide range of diseases, playing a crucial role in controlling both infectious diseases and chronic non-communicable diseases [167]. For HCMV infection, vaccine development has been a key area of research focus. Despite more than 50 years of research efforts, no licensed vaccine is currently available for HCMV. The development of an effective HCMV vaccine faces several challenges, including the complex viral life cycle, the ability of HCMV to evade host immune responses, and the lack of a clear understanding of immune correlates of protection. Nevertheless, significant progress has been made in recent years, with various vaccine strategies being explored in clinical trials. Several types of HCMV vaccines have been developed and tested, including live-attenuated vaccines, protein subunit vaccines, vectored vaccines, DNA vaccines, and more recently, mRNA vaccines. These vaccines target various HCMV antigens, with a focus on envelope glycoproteins such as gB and the pentamer complex. Additionally, some vaccine strategies have incorporated non-envelope proteins like pp65 and IE1, which are important targets for T cell responses.

V160 is a live-attenuated cytomegalovirus (CMV) vaccine developed by Merck, in which the pentameric complex has been restored on the surface of the AD169 strain. In a phase 2b clinical study evaluating the efficacy and safety of the live-attenuated CMV vaccine V160 in preventing primary CMV infection, V160 showed overall good tolerability and immunogenicity; however, compared with placebo, vaccination with three doses did not reduce the rate of primary CMV infection in CMV-negative women (Table 2). This clinical trial was registered at ClinicalTrials.gov (NCT03486834) and EudraCT (2017-004233-86) [168]. Currently, live-attenuated CMV vaccines have not been prioritized over other vaccine types due to perceived higher potential risks.

The gB/MF59 recombinant protein vaccine, composed of the squalene adjuvant MF59 and the monomeric gB produced in recombinant Chinese hamster ovary cells [169], protected “approximately half of vaccinees” from CMV infection in phase II clinical trials (NCT00125502, NCT00133497, NCT00815165). However, no further phase III trial was conducted after completion in 2013. GSK1492903A, developed by GlaxoSmithKline, is an adjuvanted subunit vaccine of glycoprotein B (gB) from the AD169 strain. This vaccine has been studied for safety and immunogenicity in CMV-negative healthy adult male subjects (NCT00435396). Additionally, GSK3993129 is a candidate CMV recombinant protein subunit (CMVsu) vaccine comprising gB and an adjuvanted pentameric antigen, which has been evaluated in healthy adults for safety and immune responses at four increasing dose levels (NCT05089630). Recent studies have focused on improving gB-based vaccines through structure-based design and the inclusion of additional antigens [170].

VCL-CT02 is a trivalent hCMV DNA vaccine in development by Vical, consisting of plasmids encoding hCMV pp65, gB, and IE1 [171]. In a phase I trial, unadjuvanted VCL-CT02 showed moderate immunogenicity; however, when subjects received VCL-CT02 containing the live-attenuated Towne hCMV strain, enhanced pp65 T-cell responses and gB antibody responses were observed compared to the Towne control alone (NCT00373412, NCT00370006).

VCL-CB01, also known as ASP0113, is a bivalent polymeric methylmethacrylate DNA vaccine developed by Vical for the prevention of HCMV infection and disease, containing plasmids encoding HCMV capsid phosphoprotein 65 and the major surface glycoprotein B [172]. A phase I trial in healthy adults showed good tolerability of VCL-CB01 (NCT02103426) [172]. Interim results from a phase II trial in HCMV-positive hematopoietic cell transplant recipients demonstrated increased T-cell responses with VCL-CB01 compared to placebo (NCT00285259) [173]. In a phase II study, the vaccine failed to outperform placebo in preventing CMV in kidney transplant patients, dashing prospects in this population (NCT01974206). Subsequently, in a phase III trial in hematopoietic stem cell transplant (HCT) recipients, the vaccine did not show a significant improvement in overall survival or reduction in CMV end-organ disease, missing the primary composite endpoint (NCT01877655). It also missed secondary endpoints, including time to first CMV and time to first antiviral therapy use [174].

Moderna has developed an mRNA-1647 vaccine against CMV, consisting of 6 mRNA sequences encoding the 2 CMV antigens glycoprotein B and the pentameric glycoprotein complex [175]. Results from a phase II, randomized, observer-blind, placebo-controlled, dose-ranging trial showed that mRNA-1647 demonstrated a favorable safety and tolerability profile in vaccinees. A subsequent study evaluated antibody responses induced by mRNA-1647 in HCMV seronegative and seropositive vaccinees from first vaccination through one year after the third vaccination using systems serology approaches [176]. In this study, the mRNA-1647 HCMV vaccine was confirmed to elicit robust and durable HCMV-specific IgG responses in seronegative vaccinees and boosted pre-existing HCMV-specific IgG responses in seropositive vaccinees. Moreover, mRNA-1647 elicited broad and potent neutralizing antibody responses as well as Fc-mediated effector functions, including robust ADCC responses. Currently, a phase III clinical study is evaluating the safety and efficacy of a 100 µg dose of mRNA-1647 against primary CMV infection in women aged 16–40 years (NCT05085366).

Vector-based vaccines have also shown promise. Several viral vectors have been explored for HCMV vaccine development, including canarypox, alphavirus replicons, and modified vaccinia Ankara (MVA). Notably, the CMV-MVA triplex vaccine, developed by researchers at City of Hope, is an attenuated poxvirus modified vaccinia Ankara vector encoding CMV pp65, IE1-exon 4, and IE2-exon5. This vaccine has been evaluated in multiple clinical trials. In a phase I study (NCT01941056), the vaccine was safe, well-tolerated and highly immunogenic in healthy adults. Currently, several phase 2 trials are ongoing, including a study assessing the vaccine’s ability to reduce CMV viremia in hematopoietic cell transplant (HCT) recipients (NCT02506933), a study in CMV-seropositive HCT recipients who discontinue letermovir prophylaxis (NCT04060277), and another evaluating the vaccine’s efficacy in CMV-seropositive adults with HIV (NCT05099965). ALVAC-CMV (vCP260) is a canarypox vector vaccine expressing HCMV pp65. In a phase II clinical trial, this vaccine was evaluated in healthy adults and HCMT donor-recipient pairs (NCT00353977).

VBI-1501 is a vaccine developed using enveloped virus-like particle (eVLP) technology, comprising a recombinant protein that fuses the ectodomain of glycoprotein B from the Towne strain of human cytomegalovirus with the transmembrane and cytoplasmic domains of the VSV G protein. Phase I results indicated that VBI-1501 was safe and well-tolerated at all dose levels [177]. 100% of subjects receiving the highest dose elicited neutralizing antibodies against fibroblast-entry of CMV, with titers comparable to those observed in naturally infected subjects with protective immunity. In the same dose group, 31% of subjects also developed neutralizing antibodies against epithelial cell entry (NCT02826798). In 2019, a phase II clinical trial was announced to recruit approximately 110 male and female subjects aged 18 to 40 years to evaluate the safety and immunogenicity of three different dose levels of VBI-1501: 5 µg, 10 µg, and 20 µg.

HB-101 is a bivalent cytomegalovirus vaccine candidate based on HOOKIPA’s proprietary arenaviral vector platform using its non-replicating VaxWave technology [178], with two arenaviral vectors containing two antigens, human cytomegalovirus phosphoprotein 65 (pp65, a T cell antigen) and glycoprotein B (gB, a B cell antigen) fusion protein, aimed at triggering T cell and B cell immunity through “infection” of antigen presenting cells. Phase II clinical data in 83 kidney transplant patients showed that compared to placebo, two doses of HB-101 did not reduce the incidence of post-transplant cytomegalovirus infection, disease or antiviral therapy, leading to the discontinuation of further development (NCT03629080).

AVX601 is a bivalent alphavirus replicon vaccine derived from Venezuelan equine encephalitis virus, in which the viral structural proteins have been replaced with genes encoding the extracellular domain of Towne gB and a pp65/IE1 fusion protein expressed from a dual promoter replicon [179]. In a phase I trial, seronegative volunteers received three low or high doses of the vaccine over 24 weeks. It was found to be safe and induced high levels of neutralizing antibodies and polyfunctional CD4^+^ and CD8^+^ antigen-specific T cell responses (NCT00439803).

**Table 2:** Selected HCMV vaccines under clinical development

Types of vaccine	Vaccine name	Description	Clinical status	Clinical trial number
Live attenuated vaccine	V160	Attenuated vaccine of HCMV AD169 with restored pentamer expression	Phase IIb	NCT03486834, EudraCT (2017-004233-86)
Protein/subunit vaccine	gB/MF59	Composition of squalene adjuvant MF59 and recombinant gB	Phase II	NCT00125502、 NCT00133497、NCT00815165
	GSK1492903A	gB of HCMV AD169	Phase I	NCT00435396
	GSK3993129	Composition of gB and pentamer	Phase I/II	NCT02472548
DNA-based vaccine	VCL-CT02	A trivalent hCMV DNA vaccine consisting of plasmids encoding hCMV pp65, gB and IE1	Phase I	NCT00373412, NCT00370006,
	VCL-CB01 (ASP0113)	A bivalent DNA vaccine encoding HCMV capsid phosphoprotein 65 and gB	Phase III	NCT01974206, NCT01877655
mRNA-based vaccine	mRNA-1647	Encoding gB and pentamer	Phase III	NCT05085366
Vector-based vaccine	CMV-MVA	Ankara vector encoding CMV pp65, IE1-exon 4, and IE2-exon5	Phase II	NCT01941056 NCT02506933 NCT04060277 NCT05099965
	vCP260	Canarypox vector vaccine expressing HCMV pp65	Phase II	NCT00353977
	VBI-1501	An enveloped virus-like particle (eVLP) vaccine expressing Towne strain gB	Phase II	NCT02826798
	HB-101	An arenavirus vector-based vaccine encoding pp65 and gB proteins	Phase II	NCT03629080
	AVX 601	A vaccine based on Venezuelan equine encephalitis virus encoding pp65/IE1 fusion protein and gB protein	Phase I	NCT00439803

### Inhibitors

HCMV inhibitors can be categorized into two main groups: those directly targeting the virus and those modulating host cellular functions to indirectly combat viral infection (Table 3).

#### Virus-targeted inhibitors

Virus-targeted inhibitors primarily interfere with viral replication by inhibiting viral DNA polymerase or other key viral proteins. Among these, nucleoside and nucleotide analogues have long been the cornerstone of anti-HCMV therapy [180]. The most commonly used compounds in this class are ganciclovir (GCV) and its oral prodrug valganciclovir (VGCV), which are first-line treatments for HCMV infections [181]. GCV is activated through multi-step phosphorylation by viral UL97 kinase and cellular kinases, and its triphosphate form competitively inhibits viral DNA polymerase and can be incorporated into viral DNA, leading to chain termination [182]. Although GCV and VGCV are highly effective, they are associated with significant toxicity, particularly bone marrow suppression, which may limit their use in some patients [183].

To overcome the limitations of GCV, researchers have developed other nucleoside and nucleotide analogues. For example, cidofovir (CDV) is an acyclic nucleotide analogue that does not require initial activation by viral kinases but is directly phosphorylated by cellular enzymes to its active form [184]. CDV’s advantage lies in its effectiveness against some GCV-resistant HCMV strains. However, its use is limited by nephrotoxicity, requiring careful monitoring of renal function during treatment [185, 186]. To further improve drug properties, researchers developed brincidofovir (BCV), a lipid conjugate of cidofovir. BCV was designed to improve oral bioavailability and reduce nephrotoxicity associated with CDV [187]. Although it showed promising antiviral activity and better safety in preclinical and early clinical studies, its development was discontinued after failing to meet the primary endpoint in a phase III clinical trial [188]. Another novel anti-HCMV compound is cyclopropavir (CPV), a methylenecyclopropane nucleoside analogue. CPV is effective not only against HCMV but also against Epstein-Barr virus (EBV) [189]. CPV is activated through phosphorylation by viral UL97 kinase and cellular enzymes, and its triphosphate form inhibits viral DNA polymerase. Interestingly, CPV also inhibits the UL97 kinase itself, providing a dual mechanism of action [190]. While promising in early clinical trials, further studies are needed to establish its efficacy and safety profile in different patient populations.

Besides nucleoside and nucleotide analogues, antiviral drugs with other mechanisms of action have also been developed. Foscarnet is an inorganic pyrophosphate analogue that inhibits viral DNA polymerase by binding to the pyrophosphate binding site [191]. Unlike nucleoside analogues, foscarnet does not require activation by viral or cellular kinases. It is often used to treat GCV-resistant HCMV infections, particularly in transplant recipients and HIV/AIDS patients. However, its use is limited by nephrotoxicity and the need for intravenous administration, which can complicate long-term treatment [192].

In recent years, the viral terminase complex has emerged as an attractive new target for anti-HCMV therapy. Letermovir is a novel antiviral drug that specifically targets the pUL56 subunit of the HCMV terminase complex [193]. By inhibiting viral DNA packaging, letermovir effectively suppresses viral replication at a stage distinct from DNA polymerase inhibitors. This unique mechanism of action allows letermovir to remain effective against HCMV strains resistant to other antiviral drugs. Letermovir has shown promising results in clinical trials and has been approved for prophylaxis in HCMV-seropositive recipients of allogeneic hematopoietic stem cell transplants [194]. Other terminase inhibitors, such as benzimidazole derivatives BDCRB, TCRB, and tomeglovir, have also shown potential in preclinical studies [195, 196]. These compounds target various components of the terminase complex, including pUL56 and pUL89. Although they have demonstrated potent anti-HCMV activity in vitro, their development has not progressed to clinical trials due to pharmacokinetic limitations and potential toxicity concerns [197].

Finally, maribavir is a novel antiviral drug that specifically inhibits the HCMV UL97 kinase [198]. This protein kinase plays crucial roles in viral replication, including phosphorylation of viral and cellular proteins involved in viral DNA synthesis and nuclear egress of viral capsids. By inhibiting UL97, maribavir interferes with multiple stages of the viral life cycle. Notably, maribavir has shown efficacy against some GCV-resistant HCMV strains, as its mechanism of action is independent of the viral DNA polymerase [199]. Recently, maribavir was approved for treating post-transplant HCMV infections refractory to other treatments, providing a valuable option for managing difficult cases [200, 201].

#### Host-targeted inhibitors

Host-targeted inhibitors exert their antiviral effects by interfering with cellular components required for viral replication. These inhibitors target different points, have varying efficacies, but share the common feature that no viral resistance to them has been identified to date.

Among these, artemisinin derivatives, such as artesunate, have shown promising results in preclinical studies and some clinical cases [202]. Artesunate acts by inhibiting cellular transcription factors Sp1 and NF-κB, which are crucial for HCMV replication [203]. It has demonstrated efficacy in in vitro studies and in some clinical cases of drug-resistant HCMV infections [204, 205]. This novel mechanism of action provides a potential treatment option for refractory HCMV infections. Similarly, flavonoid compounds, particularly quercetin and baicalein, have also shown anti-HCMV activity in in vitro studies [206]. These compounds act by inhibiting early viral protein production and modulating cellular pathways involved in viral replication. While these results are encouraging, further research is needed to determine their clinical efficacy and safety.

Cyclooxygenase-2 (COX-2) inhibitors are another class of compounds that have shown potential anti-HCMV activity in preclinical studies [207]. HCMV infection upregulates COX-2 expression, and inhibiting this enzyme appears to interfere with viral replication [208, 209]. While promising, this approach requires further research to determine its clinical relevance.

In addition to these small molecule inhibitors, some drugs originally used for other diseases have been found to have anti-HCMV effects. For example, leflunomide, an immunosuppressant used to treat rheumatoid arthritis, has demonstrated anti-HCMV effects by inhibiting viral replication through pyrimidine synthesis inhibition [210]. It has been used off-label to treat refractory HCMV infections, particularly in transplant recipients [211]. This drug repurposing strategy provides new possibilities for treating HCMV infections.

Finally, mTOR inhibitors, such as everolimus, have been associated with a reduced incidence of HCMV infection in transplant recipients [212]. These drugs are thought to enhance T cell responses against HCMV while maintaining overall immunosuppression. This dual effect makes mTOR inhibitors particularly interesting for preventing HCMV disease in transplant settings [213]. This approach not only targets the virus but also modulates the host’s immune response, providing a comprehensive approach to managing HCMV infections.

**Table 3:** Current and emerging inhibitors of human cytomegalovirus

Inhibitor	Developer/Inventor	Target	Mechanism of Action	Clinical Status
Ganciclovir (GCV)	Syntex	Viral DNA polymerase	Inhibits viral DNA replication after phosphorylation by viral UL97 kinase and cellular kinases	FDA approved
Valganciclovir (VGCV)	Roche	Viral DNA polymerase	Oral prodrug of GCV	FDA approved
Cidofovir (CDV)	Gilead Sciences	Viral DNA polymerase	Directly inhibits viral DNA polymerase after phosphorylation by cellular enzymes	FDA approved
Foscarnet	Astra	Viral DNA polymerase	Inhibits viral DNA polymerase by binding to the pyrophosphate binding site	FDA approved
Letermovir	AiCuris/Merck	Terminase complex (pUL56)	Inhibits viral DNA packaging	FDA approved
Maribavir	ViroPharma/Shire	UL97 kinase	Inhibits viral protein phosphorylation and capsid nuclear egress	FDA approved
Brincidofovir (BCV)	Chimerix	Viral DNA polymerase	Lipid conjugate of cidofovir with improved cellular uptake	Phase III (discontinued)
Cyclopropavir (CPV)	Multiple research groups	UL97 kinase and DNA polymerase	Inhibits viral DNA replication and UL97 kinase	Phase I
BDCRB/TCRB	Townsend et al.	Terminase complex	Inhibits viral DNA packaging	Preclinical
Tomeglovir	Bayer	Terminase complex	Inhibits viral DNA packaging	Preclinical
Leflunomide	Sanofi	Pyrimidine synthesis	Inhibits viral replication	Off-label use
Everolimus	Novartis	mTOR	Enhances T cell response against HCMV	FDA approved (prophylaxis)
Artesunate	Multiple research groups	NF-κB signaling pathway	Inhibits viral replication	Phase III
COX-2 inhibitors	Multiple research groups	COX-2	Inhibits viral replication	Preclinical
Quercetin	Natural compound	Multiple host targets	Modulates host cell signaling	Preclinical
Baicalein	Natural compound	Multiple host targets	Modulates host cell signaling	Preclinical

### TCR-T cell therapy

T cell receptor-engineered T (TCR-T) cell therapy has emerged as a promising approach for treating CMV infections and CMV-associated malignancies. Compared to other cellular therapies like chimeric antigen receptor (CAR) T cells, TCR-T cells offer several advantages for targeting CMV. TCR-T cells can recognize intracellular antigens presented by MHC molecules, allowing for a broader range of targetable epitopes. Additionally, the use of naturally occurring TCRs may reduce the risk of off-target effects [214, 215].

Recent studies have demonstrated the efficacy of CMV-specific TCR-T cells in controlling CMV infections post-hematopoietic stem cell transplantation (HSCT). In a phase I clinical trial (NCT04153279), pp65-specific TCR-T cell therapy showed encouraging results in treating CMV reactivation after HSCT, with most patients achieving complete viral clearance and the TCR-T cells persisting for up to 3 months [216]. Liu et al. further elaborated on this trial, reporting that six out of seven patients achieved complete response (CR) with no more than grade 2 cytokine release syndrome (CRS) and other adverse events observed. Notably, CMV TCR-T cells persisted up to 3 months, and two patients survived for more than 1 year [216]. This approach offers advantages over conventional antiviral drugs, including reduced toxicity and the potential for long-term protection against CMV reactivation.

The development of CMV-specific TCR-T cells has been facilitated by advances in single-cell sequencing technologies and bioinformatics tools. Researchers have employed single-cell TCR sequencing and algorithms like GLIPH2 (Grouping of Lymphocyte Interactions by Paratope Hotspots) to identify and characterize CMV-specific TCR sequences from patients [217]. CMV-specific TCR-T cells have also shown promise in treating CMV-associated malignancies, such as glioblastoma (GBM). In preclinical studies, CMV-TCR-T cells demonstrated significant antitumor activity against CMV-infected GBM cells both in vitro and in orthotopic mouse models [217]. These findings suggest that CMV-TCR-T cell therapy could potentially address the limitations of current immunotherapies in treating CMV-positive GBMs.

Recent advancements in mRNA technology have further improved the potential of TCR-T cell therapy. Shen et al. introduced the use of circular mRNA (cmRNA) for TCR-T cell production, demonstrating its superiority over linear mRNA due to higher resistance to exonuclease degradation, resulting in greater stability and protein expression [218]. Their study showed that monocyte-derived dendritic cells transfected with pp65-encoding cmRNA were more effective in expanding pp65-responsive T cells compared to linear mRNA. The cmRNA-transduced pp65-TCR-T (cm-pp65-TCR-T) cells demonstrated specific targeting of the CMV-pp65 epitope, with TCR expression on primary T cells lasting for more than 7 days. Both in vitro killing assays and in vivo CDX models showed that cm-pp65-TCR-T cells specifically and persistently killed pp65- and HLA-expressing tumor cells, significantly prolonging the survival of mice [218].

## Summary and outlook

The prevention and treatment of human cytomegalovirus (HCMV) infection remain a key challenge in modern medical research. This review has delved into HCMV entry mechanisms, immune evasion strategies, and current preventive and therapeutic approaches, providing important insights for future research directions.

Regarding HCMV entry mechanisms, recent studies have revealed the complexity of interactions between viral envelope glycoproteins and host cell receptors. Glycoproteins gB, the gH/gL/gO trimer, and the gH/gL/pUL128-131 pentamer play crucial roles in viral attachment and entry. Notably, the recently resolved high-resolution structure of the gH/gL/gO-PDGFRα complex has provided new insights into HCMV receptor recognition. However, many questions remain unanswered, such as the exact identity of the gB fusion protein receptor, the molecular mechanisms triggering gB conformational changes, and the differences in HCMV entry pathways across various cell types. Future research needs to further elucidate the structure-function relationships of these key proteins, providing a foundation for developing new therapeutic strategies.

HCMV’s immune evasion strategies demonstrate the result of long-term co-evolution between the virus and the host immune system. The virus interferes with both innate and adaptive immune responses through multiple mechanisms, including suppression of pattern recognition receptor signaling pathways, regulation of interferon production and signaling, modulation of NK cell activity, interference with antigen presentation, and regulation of T and B cell functions. These complex immune evasion strategies allow HCMV to establish persistent infection and long-term presence in the host. Future research needs to delve deeper into the molecular basis of these immune evasion mechanisms and how they coordinate with each other. This knowledge will aid in developing more effective immunotherapeutic strategies, such as methods to enhance specific T cell responses or targeted drugs to block key immune evasion pathways.

In terms of prevention and treatment, significant progress has been made in recent years in the development of antibodies and vaccines targeting key HCMV surface proteins. Monoclonal antibodies targeting gB, gH/gL, and the pentamer have shown promising neutralizing activity. However, since HCMV utilizes multiple surface proteins for invasion, a single antibody may struggle to completely block infection. Future research directions may include developing antibody cocktails or bispecific antibodies targeting multiple epitopes to enhance neutralization potency and breadth. In vaccine development, exploration continues from traditional inactivated and attenuated vaccines to new subunit, vector-based, and mRNA vaccines. Among these, Moderna’s mRNA-1647 vaccine has shown promising results in clinical trials, drawing attention to the potential of mRNA vaccine technology in HCMV prevention. However, developing a vaccine that effectively prevents HCMV infection still faces many challenges, including how to induce lasting humoral and cellular immune responses and how to overcome HCMV’s immune evasion mechanisms. Future directions in HCMV vaccine development may include multivalent vaccine approaches, novel adjuvant formulations, exploration of new vaccine delivery platforms, and improved animal models that better recapitulate human HCMV infection and immune responses. In the field of antiviral drugs, besides traditional nucleoside analogues like ganciclovir, the emergence of new drugs with novel mechanisms of action, such as letermovir (targeting the terminase complex) and maribavir (targeting UL97 kinase), has provided new treatment options for refractory HCMV infections. However, drug resistance and toxicity remain challenges to be overcome. Future research may focus more on host-targeted inhibitors, such as compounds that modulate cellular signaling pathways or metabolism, to provide new therapeutic strategies. TCR-T cell therapy, as an emerging treatment method, shows promising prospects in controlling HCMV infection and treating HCMV-associated malignancies. Compared to CAR-T cells, TCR-T cells can recognize intracellular antigens presented by MHC molecules, offering a broader range of targeting possibilities. However, this approach still requires further research to determine its long-term safety and efficacy, as well as how to overcome the impact of HCMV’s immune evasion mechanisms on T cell function.

Overall, the field of HCMV research is at an exciting juncture, with new molecular and structural insights driving the development of innovative therapeutic strategies. In the future, interdisciplinary research approaches will become increasingly important, combining knowledge from virology, immunology, structural biology, and drug development to develop more effective HCMV prevention and treatment methods. In particular, a deep understanding of HCMV’s entry mechanisms and immune evasion strategies will provide crucial guidance for developing a new generation of antibodies, vaccines, and small molecule inhibitors with stronger targeting and better efficacy. Meanwhile, the development of personalized treatment strategies, such as precision treatment plans based on patient immune status and viral strain characteristics, may also become an important direction for future research.

Finally, considering the severe impact of HCMV infection on immunocompromised patients and congenitally infected newborns, future research needs to pay more attention to these high-risk populations and develop targeted prevention and treatment strategies. This not only requires breakthroughs at the basic research level but also more clinical trials to verify the effectiveness and safety of new methods. Only through continued scientific investment and multifaceted efforts can we ultimately achieve effective control of HCMV infection and reduce its impact on public health.

## Supplementary Information

Supplementary Material 1.

Supplementary Material 2.

Supplementary Material 3.

Supplementary Material 4.

Supplementary Material 5.

Supplementary Material 6.

Supplementary Material 7.

## Data Availability

The sequence data used for our analyses are as follows: The multiple sequence alignments used for conservation analysis of HCMV glycoproteins were generated using publicly available sequences from GenBank (https://www.ncbi.nlm.nih.gov/genbank/). The specific sequences used include: gB protein sequences (*n* = 198). gH protein sequences (*n* = 85). gL protein sequences (*n* = 102). gO protein sequences (*n* = 128). UL128 protein sequences (*n* = 74). UL130 protein sequences (*n* = 147). UL131 protein sequences (*n* = 42). UL116 protein sequences (*n* = 216). The complete sequence datasets, including accession numbers and multiple sequence alignments used for conservation analysis in Sect. 3.4, are provided as Supplementary Data 1.

## References

[1] Goodrum F, Caviness K, Zagallo P. Human cytomegalovirus persistence. Cell Microbiol. 2012;14(5):644–55. 10.1111/j.1462-5822.2012.01774.x. 22329758 10.1111/j.1462-5822.2012.01774.x PMC3330195

[2] Britt WJ. Maternal immunity and the natural history of congenital human cytomegalovirus infection. Viruses. 2018;10(8): 405. 10.3390/v10080405. 30081449 10.3390/v10080405 PMC6116058

[3] Messinger CJ, Lipsitch M, Bateman BT, He M, Huybrechts KF, MacDonald S, et al. Association between congenital Cytomegalovirus and the prevalence at birth of microcephaly in the United States. JAMA Pediatr. 2020;174(12):1159–67. 10.1001/jamapediatrics.2020.3009. 32926077 10.1001/jamapediatrics.2020.3009 PMC7490747

[4] Goderis J, De Leenheer E, Smets K, Van Hoecke H, Keymeulen A, Dhooge I. Hearing loss and congenital CMV infection: a systematic review. Pediatrics. 2014;134(5):972–82. 10.1542/peds.2014-1173. 25349318 10.1542/peds.2014-1173

[5] Megli CJ, Coyne CB. Infections at the maternal–fetal interface: an overview of pathogenesis and defence. Nat Rev Microbiol. 2022;20(2):67–82. 10.1038/s41579-021-00610-y. 34433930 10.1038/s41579-021-00610-y PMC8386341

[6] Prateek B, Narang A, Minz RW. Neonatal cytomegalovirus infection: diagnostic modalities available for early disease detection. Indian J Pediatr. 2010;77(1):77–9. 10.1007/s12098-009-0255-2. 19936660 10.1007/s12098-009-0255-2

[7] Li Z, Pang J, Dong L, Yu X. Structural basis for genome packaging, retention, and ejection in human cytomegalovirus. Nat Commun. 2021;12(1):4538. 10.1038/s41467-021-24820-3. 34315863 10.1038/s41467-021-24820-3 PMC8316551

[8] Griffiths P, Reeves M. Pathogenesis of human cytomegalovirus in the immunocompromised host. Nat Rev Microbiol. 2021. 10.1038/s41579-021-00582-z. 34168328 10.1038/s41579-021-00582-z PMC8223196

[9] Wrapp D, Ye X, Ku Z, Su H, Jones HG, Wang N, et al. Structural basis for HCMV Pentamer recognition by neuropilin 2 and neutralizing antibodies. Sci Adv. 2022;8(10): eabm2546. 10.1126/sciadv.abm2546. 35275718 10.1126/sciadv.abm2546 PMC8916728

[10] Kschonsak M, Johnson MC, Schelling R, Green EM, Rougé L, Ho H, et al. Structural basis for HCMV pentamer receptor recognition and antibody neutralization. Sci Adv. 2022;8(10): eabm2536. 10.1126/sciadv.abm2536. 35275719 10.1126/sciadv.abm2536 PMC8916737

[11] Kschonsak M, Rougé L, Arthur CP, Hoangdung H, Patel N, Kim I, et al. Structures of HCMV trimer reveal the basis for receptor recognition and cell entry. Cell. 2021;184(5):1232-44.e16. 10.1016/j.cell.2021.01.036. 33626330 10.1016/j.cell.2021.01.036

[12] Liu Y, Heim KP, Che Y, Chi X, Qiu X, Han S, et al. Prefusion structure of human cytomegalovirus glycoprotein B and structural basis for membrane fusion. Sci Adv. 2021;7(10):eabf3178. 10.1126/sciadv.abf3178. 33674318 10.1126/sciadv.abf3178 PMC7935361

[13] Khanna R, Diamond DJ. Human cytomegalovirus vaccine: time to look for alternative options. Trends Mol Med. 2006;12(1):26–33. 10.1016/j.molmed.2005.11.006. 16337831 10.1016/j.molmed.2005.11.006

[14] Griffiths P, Reeves M. Pathogenesis of human cytomegalovirus in the immunocompromised host. Nat Rev Microbiol. 2021;19(12):759–73. 10.1038/s41579-021-00582-z. 34168328 10.1038/s41579-021-00582-z PMC8223196

[15] Charles OJ, Venturini C, Gantt S, Atkinson C, Griffiths P, Goldstein RA, et al. Genomic and geographical structure of human cytomegalovirus. Proceedings of the National Academy of Sciences. 2023;120(30):e2221797120. 10.1073/pnas.2221797120. 10.1073/pnas.2221797120 PMC10372631 37459519

[16] Dunn W, Chou C, Li H, Hai R, Patterson D, Stolc V, et al. Functional profiling of a human cytomegalovirus genome. Proceed National Acad Sci. 2003;100(24):14223–8. 10.1073/pnas.2334032100. 10.1073/pnas.2334032100 PMC283573 14623981

[17] Stern-Ginossar N, Weisburd B, Michalski A, Le VT, Hein MY, Huang SX, et al. Decoding human cytomegalovirus. Science. 2012;338(6110):1088–93. 10.1126/science.1227919. 23180859 10.1126/science.1227919 PMC3817102

[18] Isomura H, Stinski MF. Coordination of late gene transcription of human cytomegalovirus with viral DNA synthesis: recombinant viruses as potential therapeutic vaccine candidates. Expert Opin Ther Targets. 2013;17(2):157–66. 10.1517/14728222.2013.740460. 23231449 10.1517/14728222.2013.740460

[19] Kalejta RF. Tegument proteins of human cytomegalovirus. Microbiol Mol Biol Rev. 2008;72(2):249–65. 10.1128/MMBR.00040-07. 18535146 10.1128/MMBR.00040-07 PMC2415745

[20] Saffert RT, Kalejta RF. Inactivating a Cellular Intrinsic Immune Defense mediated by Daxx is the mechanism through which the human cytomegalovirus pp71 protein stimulates viral Immediate-Early Gene expression. J Virol. 2006;80(8):3863–71. 10.1128/jvi.80.8.3863-3871.2006. 16571803 10.1128/JVI.80.8.3863-3871.2006 PMC1440479

[21] Charman M, Weitzman MD. Replication compartments of DNA viruses in the nucleus: location, location, location. Viruses. 2020;12(2): 151. 10.3390/v12020151. 32013091 10.3390/v12020151 PMC7077188

[22] McCormick D, Lin YT, Grey F. Identification of host factors involved in human cytomegalovirus replication, assembly, and egress using a two-step small interfering RNA screen. mBio. 2018;9(3). 10.1128/mBio.00716-18. 10.1128/mBio.00716-18 PMC6020295 29946045

[23] Muller C, Alain S, Baumert TF, Ligat G, Hantz S. Structures and divergent mechanisms in capsid maturation and stabilization following genome packaging of human cytomegalovirus and herpesviruses. Life. 2021;11(2): 150. 10.3390/life11020150. 33669389 10.3390/life11020150 PMC7920273

[24] Marschall M, Muller YA, Diewald B, Sticht H, Milbradt J. The human cytomegalovirus nuclear egress complex unites multiple functions: recruitment of effectors, nuclear envelope rearrangement, and docking to nuclear capsids. Rev Med Virol. 2017;27(4): e1934. 10.1002/rmv.1934. 10.1002/rmv.1934 28664574

[25] Das S, Ortiz DA, Gurczynski SJ, Khan F, Pellett PE. Identification of human cytomegalovirus genes important for biogenesis of the cytoplasmic virion assembly complex. J Virol. 2014;88(16):9086–99. 10.1128/JVI.01141-14. 24899189 10.1128/JVI.01141-14 PMC4136295

[26] Monti CE, Mokry RL, Schumacher ML, Dash RK, Terhune SS. Computational modeling of protracted HCMV replication using genome substrates and protein temporal profiles. Proc Natl Acad Sci. 2022;119(35):e2201787119. 10.1073/pnas.2201787119. 35994667 10.1073/pnas.2201787119 PMC9437303

[27] Nguyen CC, Kamil JP. Pathogen at the gates: human cytomegalovirus entry and cell tropism. Viruses. 2018;10(12): 704. 10.3390/v10120704. 30544948 10.3390/v10120704 PMC6316194

[28] Goodrum F. Human cytomegalovirus latency: approaching the Gordian knot. Annu Rev Virol. 2016;3(1):333–57. 10.1146/annurev-virology-110615-042422. 27501258 10.1146/annurev-virology-110615-042422 PMC5514425

[29] Foglierini M, Marcandalli J, Perez L. HCMV envelope glycoprotein diversity demystified. Front Microbiol. 2019;10:10. 10.3389/fmicb.2019.01005. 31156572 10.3389/fmicb.2019.01005 PMC6529531

[30] Li G, Nguyen CC, Ryckman BJ, Britt WJ, Kamil JP. A viral regulator of glycoprotein complexes contributes to human cytomegalovirus cell tropism. Proc Natl Acad Sci U S A. 2015;112(14):4471–6. 10.1073/pnas.1419875112. 25831500 10.1073/pnas.1419875112 PMC4394275

[31] Gardner TJ, Tortorella D. Virion glycoprotein-mediated immune evasion by human cytomegalovirus: a sticky virus makes a slick getaway. Microbiol Mol Biol Rev. 2016;80(3):663–77. 10.1128/mmbr.00018-16. 27307580 10.1128/MMBR.00018-16 PMC4981668

[32] Vanarsdall AL, Johnson DC. Human cytomegalovirus entry into cells. Curr Opin Virol. 2012;2(1):37–42. 10.1016/j.coviro.2012.01.001. 22440964 10.1016/j.coviro.2012.01.001 PMC3880194

[33] Jackson SE, Mason GM, Wills MR. Human cytomegalovirus immunity and immune evasion. Virus Res. 2011;157(2):151. 10.1016/j.virusres.2010.10.031. 21056604 10.1016/j.virusres.2010.10.031

[34] Cimato G, Zhou X, Brune W, Frascaroli G. Human cytomegalovirus glycoprotein variants governing viral tropism and syncytium formation in epithelial cells and macrophages. J Virol. 2024;0(0):e00293-00224. 10.1128/jvi.00293-24. 10.1128/jvi.00293-24 PMC11265420 38837351

[35] Shimamura M, Mach M, Britt WJ. Human cytomegalovirus infection elicits a glycoprotein M (gM)/gN-Specific virus-neutralizing antibody response. J Virol. 2006;80(9):4591–600. 10.1128/jvi.80.9.4591-4600.2006. 16611919 10.1128/JVI.80.9.4591-4600.2006 PMC1471997

[36] Compton T. Receptors and immune sensors: the complex entry path of human cytomegalovirus. Trends Cell Biol. 2004;14(1):5–8. 10.1016/j.tcb.2003.10.009. 14729174 10.1016/j.tcb.2003.10.009

[37] Compton T, Nowlin DM, Cooper NR. Initiation of human cytomegalovirus infection requires initial interaction with cell surface Heparan Sulfate. Virology. 1993;193(2):834–41. 10.1006/viro.1993.1192. 8384757 10.1006/viro.1993.1192

[38] Krzyzaniak M, Mach M, Britt WJ. The cytoplasmic tail of glycoprotein M (gpUL100) expresses trafficking signals required for human Cytomegalovirus Assembly and Replication. J Virol. 2007;81(19):10316–28. 10.1128/jvi.00375-07. 17626081 10.1128/JVI.00375-07 PMC2045486

[39] Mitra D, Hasan MH, Bates JT, Bierdeman MA, Ederer DR, Parmar RC, et al. The degree of polymerization and sulfation patterns in heparan sulfate are critical determinants of cytomegalovirus entry into host cells. PLoS Pathog. 2021;17(8): e1009803. 10.1371/journal.ppat.1009803. 34352038 10.1371/journal.ppat.1009803 PMC8384199

[40] Wang X, Huong S-M, Chiu ML, Raab-Traub N, Huang E-S. Epidermal growth factor receptor is a cellular receptor for human cytomegalovirus. Nature. 2003;424(6947):456–61. 10.1038/nature01818. 12879076 10.1038/nature01818

[41] Chan G, Nogalski MT, Yurochko AD. Activation of EGFR on monocytes is required for human cytomegalovirus entry and mediates cellular motility. Proc Natl Acad Sci. 2009;106(52):22369–74. 10.1073/pnas.0908787106. 20018733 10.1073/pnas.0908787106 PMC2799688

[42] Smith MS, Bentz GL, Smith PM, Bivins ER, Yurochko AD. HCMV activates PI(3)K in monocytes and promotes monocyte motility and transendothelial migration in a PI(3)K-dependent manner. J Leukoc Biol. 2004;76(1):65–76. 10.1189/jlb.1203621. 15107461 10.1189/jlb.1203621

[43] Soroceanu L, Akhavan A, Cobbs CS. Platelet-derived growth factor-α receptor activation is required for human cytomegalovirus infection. Nature. 2008;455(7211):391–5. 10.1038/nature07209. 18701889 10.1038/nature07209

[44] Diaferia GR, Jimenez-Caliani AJ, Ranjitkar P, Yang W, Hardiman G, Rhodes CJ, et al. β1 integrin is a crucial regulator of pancreatic β-cell expansion. Development. 2013;140(16):3360–72. 10.1242/dev.098533. 23863477 10.1242/dev.098533 PMC3737718

[45] Mahmud J, Miller MJ, Altman AM, Chan GC. Human cytomegalovirus glycoprotein-initiated signaling mediates the aberrant activation of Akt. J Virol. 2020;94(16):10. 10.1128/jvi.00167-20. 10.1128/JVI.00167-20 PMC7394886 32493823

[46] Si Z, Zhang J, Shivakoti S, Atanasov I, Tao C-L, Hui WH, et al. Different functional states of fusion protein gB revealed on human cytomegalovirus by cryo electron tomography with Volta phase plate. PLoS Pathog. 2018;14(12): e1007452. 10.1371/journal.ppat.1007452. 30507948 10.1371/journal.ppat.1007452 PMC6307773

[47] Dong X-D, Li Y, Li Y, Sun C, Liu S-X, Duan H, et al. EphA2 is a functional entry receptor for HCMV infection of glioblastoma cells. PLoS Pathog. 2023;19(5): e1011304. 10.1371/journal.ppat.1011304. 37146061 10.1371/journal.ppat.1011304 PMC10191332

[48] Martinez-Martin N, Marcandalli J, Huang CS, Arthur CP, Perotti M, Foglierini M, et al. An unbiased screen for human cytomegalovirus identifies Neuropilin-2 as a central viral receptor. Cell. 2018;174(5):1158-71.e19. 30057110 10.1016/j.cell.2018.06.028

[49] Bonner JC. Mesenchymal cell survival in airway and interstitial pulmonary fibrosis. Fibrogenesis Tissue Repair. 2010;3(1): 15. 10.1186/1755-1536-3-15. 20738867 10.1186/1755-1536-3-15 PMC2940818

[50] Boström H, Willetts K, Pekny M, Levéen P, Lindahl P, Hedstrand H, et al. PDGF-A signaling is a critical event in Lung Alveolar Myofibroblast Development and Alveogenesis. Cell. 1996;85(6):863–73. 10.1016/S0092-8674(00)81270-2. 8681381 10.1016/s0092-8674(00)81270-2

[51] Grismaldo A, Sobrevia L, Morales L. Role of platelet-derived growth factor c on endothelial dysfunction in cardiovascular diseases. Biochimica et Biophysica Acta (BBA) - general subjects. 2022;1866(10): 130188. 10.1016/j.bbagen.2022.130188. 35691459 10.1016/j.bbagen.2022.130188

[52] Pellet-Many C, Frankel P, Evans Ian M, Herzog B, Jünemann-Ramírez M, Zachary Ian C. Neuropilin-1 mediates PDGF stimulation of vascular smooth muscle cell migration and signalling via p130Cas. Biochem J. 2011;435(3):609–18. 10.1042/bj20100580. 21306301 10.1042/BJ20100580 PMC3086270

[53] Giri H, Panicker SR, Cai X, Biswas I, Weiler H, Rezaie AR. Thrombomodulin is essential for maintaining quiescence in vascular endothelial cells. Proceedings of the National Academy of Sciences. 2021;118(11):e2022248118. 10.1073/pnas.2022248118. 10.1073/pnas.2022248118 PMC7980409 33836597

[54] Cohen CT, Turner NA, Moake JL. Human endothelial cells and fibroblasts express and produce the coagulation proteins necessary for thrombin generation. Sci Rep. 2021;11(1):21852. 10.1038/s41598-021-01360-w. 34750441 10.1038/s41598-021-01360-w PMC8575941

[55] Cenni E, Ciapetti G, Granchi D, Savarino L, Corradini A, Vancini M, et al. Thrombomodulin expression in endothelial cells after contact with bone cement. Biomaterials. 2002;23(10):2159–65. 10.1016/S0142-9612(01)00347-7. 11962657 10.1016/s0142-9612(01)00347-7

[56] Won T, Wood MK, Hughes DM, Talor MV, Ma Z, Schneider J, et al. Endothelial thrombomodulin downregulation caused by hypoxia contributes to severe infiltration and coagulopathy in COVID-19 patient lungs. EBioMedicine. 2022;75:75. 10.1016/j.ebiom.2022.103812. 10.1016/j.ebiom.2022.103812 PMC8756077 35033854

[57] Kabanova A, Marcandalli J, Zhou T, Bianchi S, Baxa U, Tsybovsky Y, et al. Platelet-derived growth factor-α receptor is the cellular receptor for human cytomegalovirus gHgLgO trimer. Nat Microbiol. 2016;1(8):16082. 10.1038/nmicrobiol.2016.82. 27573107 10.1038/nmicrobiol.2016.82 PMC4918640

[58] Wang J, Li J, Yin L, Pu T, Wei J, Karthikeyan V, et al. Neuropilin-2 promotes lineage plasticity and progression to neuroendocrine prostate cancer. Oncogene. 2022;41(37):4307–17. 10.1038/s41388-022-02437-0. 35986103 10.1038/s41388-022-02437-0 PMC9464715

[59] Wild JRL, Staton CA, Chapple K, Corfe BM. Neuropilins: expression and roles in the epithelium. Int J Exp Pathol. 2012;93(2):81–103. 10.1111/j.1365-2613.2012.00810.x. 22414290 10.1111/j.1365-2613.2012.00810.x PMC3385701

[60] Stein KR, Gardner TJ, Hernandez RE, Kraus TA, Duty JA, Ubarretxena-Belandia I, et al. CD46 facilitates entry and dissemination of human cytomegalovirus. Nat Commun. 2019;10(1):2699. 10.1038/s41467-019-10587-1. 31221976 10.1038/s41467-019-10587-1 PMC6586906

[61] Meraner EX, Lu P, Perreira P, Aker JM, McDougall AM. OR14I1 is a receptor for the human cytomegalovirus pentameric complex and defines viral epithelial cell tropism. Proc Natl Acad Sci U S A. 2019;116(14):7043–52. 10.1073/pnas.1814850116. 30894498 10.1073/pnas.1814850116 PMC6452726

[62] Vanarsdall AL, Pritchard SR, Wisner TW, Liu J, Jardetzky TS, Johnson DC. CD147 promotes entry of pentamer-expressing human cytomegalovirus into epithelial and endothelial cells. mBio. 2018;9(3). 10.1128/mBio.00781-18. 10.1128/mBio.00781-18 PMC5941078 29739904

[63] Wang K, Chen W, Zhang Z, Deng Y, Lian J-Q, Du P, et al. CD147-spike protein is a novel route for SARS-CoV-2 infection to host cells. Signal Transduct Target Therapy. 2020;5(1):283. 10.1038/s41392-020-00426-x. 10.1038/s41392-020-00426-x PMC7714896 33277466

[64] Ragotte RJ, Pulido D, Donnellan FR, Hill ML, Gorini G, Davies H, et al. Human basigin (CD147) does not directly interact with SARS-CoV-2 spike glycoprotein. mSphere. 2021;6(4). 10.1128/msphere.00647. 10.1128/mSphere.00647-21 PMC8386461 34378982

[65] Pushkarsky T, Zybarth G, Dubrovsky L, Yurchenko V, Tang H, Guo H, et al. CD147 facilitates HIV-1 infection by interacting with virus-associated cyclophilin A. Proceedings of the National Academy of Sciences. 2001;98(11):6360–5. 10.1073/pnas.111583198. 10.1073/pnas.111583198 PMC33473 11353871

[66] Watanabe A, Yoneda M, Ikeda F, Terao-Muto Y, Sato H, Kai C. CD147/EMMPRIN acts as a functional entry receptor for Measles Virus on epithelial cells. J Virol. 2010;84(9):4183–93. 10.1128/jvi.02168-09. 20147391 10.1128/JVI.02168-09 PMC2863760

[67] Caló S, Cortese M, Ciferri C, Bruno L, Gerrein R, Benucci B, et al. The human cytomegalovirus *UL116* gene encodes an envelope glycoprotein forming a complex with gH independently from gL. J Virol. 2016;90(10):4926–38. 10.1128/jvi.02517-15. 26937030 10.1128/JVI.02517-15 PMC4859709

[68] Siddiquey MNA, Schultz EP, Yu Q, Amendola D, Vezzani G, Yu D, et al. The human cytomegalovirus protein UL116 interacts with the viral endoplasmic-reticulum-resident glycoprotein UL148 and promotes the incorporation of gH/gL complexes into virions. J Virol. 2021;95(15). doi: 10.1128/jvi.02207-20. 34011552 10.1128/JVI.02207-20 PMC8274611

[69] Atalay R, Zimmermann A, Wagner M, Borst E, Benz C, Messerle M, et al. Identification and expression of human cytomegalovirus transcription units coding for two distinct Fcγ receptor homologs. J Virol. 2002;76(17):8596–608. 10.1128/jvi.76.17.8596-8608.2002. 12163579 10.1128/JVI.76.17.8596-8608.2002 PMC136976

[70] Cortese M, Calò S, D’Aurizio R, Lilja A, Pacchiani N, Merola M. Recombinant human cytomegalovirus (HCMV) RL13 binds human Immunoglobulin G Fc. PLoS ONE. 2012;7(11):e50166. 10.1371/journal.pone.0050166. 23226246 10.1371/journal.pone.0050166 PMC3511460

[71] Ndjamen B, Joshi DS, Fraser SE, Bjorkman PJ. Characterization of antibody bipolar bridging mediated by the human cytomegalovirus fc receptor gp68. J Virol. 2016;90(6):3262–7. 10.1128/jvi.02855-15. 26739053 10.1128/JVI.02855-15 PMC4810659

[72] Hetzenecker S, Helenius A, Krzyzaniak MA. HCMV induces macropinocytosis for host cell entry in fibroblasts. Traffic. 2016;17(4):351–68. 10.1111/tra.12355. 26650385 10.1111/tra.12355

[73] Li Q, Wilkie AR, Weller M, Liu X, Cohen JI. THY-1 cell Surface Antigen (CD90) has an important role in the initial stage of human cytomegalovirus infection. PLoS Pathog. 2015;11(7): e1004999. 10.1371/journal.ppat.1004999. 26147640 10.1371/journal.ppat.1004999 PMC4492587

[74] Li Q, Fischer E, Cohen JI. Cell surface THY-1 contributes to human cytomegalovirus entry via a macropinocytosis-like process. J Virol. 2016;90(21):9766–81. 10.1128/jvi.01092-16. 27558416 10.1128/JVI.01092-16 PMC5068528

[75] Mach M, Kropff B, Kryzaniak M, Britt W. Complex formation by glycoproteins M and N of Human Cytomegalovirus: structural and functional aspects. J Virol. 2005;79(4):2160–70. 10.1128/jvi.79.4.2160-2170.2005. 15681419 10.1128/JVI.79.4.2160-2170.2005 PMC546557

[76] Chandramouli S, Ciferri C, Nikitin PA, Caló S, Gerrein R, Balabanis K, et al. Structure of HCMV glycoprotein B in the postfusion conformation bound to a neutralizing human antibody. Nat Commun. 2015;6(1):8176. 10.1038/ncomms9176. 26365435 10.1038/ncomms9176 PMC4579600

[77] Renzette N, Gibson L, Bhattacharjee B, Fisher D, Schleiss MR, Jensen JD, et al. Rapid Intrahost evolution of human cytomegalovirus is shaped by demography and positive selection. PLoS Genet. 2013;9(9): e1003735. 10.1371/journal.pgen.1003735. 24086142 10.1371/journal.pgen.1003735 PMC3784496

[78] Bagdonaite I, Nordén R, Joshi HJ, King SL, Vakhrushev SY, Olofsson S, et al. Global mapping of O-glycosylation of varicella zoster virus, human cytomegalovirus, and epstein-barr virus. J Biol Chem. 2016;291(23):12014–28. 10.1074/jbc.M116.721746. 27129252 10.1074/jbc.M116.721746 PMC4933254

[79] Chandramouli S, Malito E, Nguyen T, Luisi K, Donnarumma D, Xing Y, et al. Structural basis for potent antibody-mediated neutralization of human cytomegalovirus. Sci Immunol. 2017;2(12): eaan1457. 10.1126/sciimmunol.aan1457. 28783665 10.1126/sciimmunol.aan1457

[80] Huang ZF, Zou HM, Liao BW, Zhang HY, Yang Y, Fu YZ, et al. Human cytomegalovirus protein UL31 inhibits DNA sensing of cGAS to Mediate Immune Evasion. Cell Host Microbe. 2018;24(1):69-80.e4. 10.1016/j.chom.2018.05.007. 29937271 10.1016/j.chom.2018.05.007

[81] Fu YZ, Guo Y, Zou HM, Su S, Wang SY, Yang Q, et al. Human cytomegalovirus protein UL42 antagonizes cGAS/MITA-mediated innate antiviral response. PLoS Pathog. 2019;15(5): e1007691. 10.1371/journal.ppat.1007691. 31107917 10.1371/journal.ppat.1007691 PMC6527189

[82] Biolatti M, Dell’Oste V, Pautasso S, Gugliesi F, von Einem J, Krapp C, et al. Human cytomegalovirus tegument protein pp65 (pUL83) dampens type I interferon production by inactivating the DNA sensor cGAS without affecting STING. J Virol. 2018;92(6):01774–01717. 10.1128/jvi. 10.1128/JVI.01774-17 PMC5827387 29263269

[83] Fu YZ, Su S, Gao YQ, Wang PP, Huang ZF, Hu MM, et al. Human cytomegalovirus tegument protein UL82 inhibits STING-Mediated signaling to evade antiviral immunity. Cell Host Microbe. 2017;21(2):231–43. 10.1016/j.chom.2017.01.001. 28132838 10.1016/j.chom.2017.01.001

[84] Zou HM, Huang ZF, Yang Y, Luo WW, Wang SY, Luo MH, et al. Human cytomegalovirus protein UL94 targets MITA to evade the antiviral immune response. J Virol. 2020;94(12). 10.1128/jvi.00022−20. 10.1128/JVI.00022-20 PMC7307088 32238587

[85] Ren Y, Wang A, Wu D, Wang C, Huang M, Xiong X, et al. Dual inhibition of innate immunity and apoptosis by human cytomegalovirus protein UL37 × 1 enables efficient virus replication. Nat Microbiol. 2022;7(7):1041–53. 10.1038/s41564-022-01136-6. 35637330 10.1038/s41564-022-01136-6

[86] Landais I, Pelton C, Streblow D, DeFilippis V, McWeeney S, Nelson JA. Human cytomegalovirus miR-UL112-3p targets TLR2 and modulates the TLR2/IRAK1/NFκB signaling pathway. PLoS Pathog. 2015;11(5): e1004881. 10.1371/journal.ppat.1004881. 25955717 10.1371/journal.ppat.1004881 PMC4425655

[87] Park A, Ra EA, Lee TA, Choi HJ, Lee E, Kang S, et al. HCMV-encoded US7 and US8 act as antagonists of innate immunity by distinctively targeting TLR-signaling pathways. Nat Commun. 2019;10(1):4670. 10.1038/s41467-019-12641-4. 31604943 10.1038/s41467-019-12641-4 PMC6789044

[88] Kim J-E, Kim Y-E, Stinski MF, Ahn J-H, Song Y-J. Human cytomegalovirus IE2 86 kDa protein induces STING degradation and inhibits cGAMP-mediated IFN-β induction. Front Microbiol. 2017;8: 1854. 10.3389/fmicb.2017.01854. 29018427 10.3389/fmicb.2017.01854 PMC5622937

[89] Choi HJ, Park A, Kang S, Lee E, Lee TA, Ra EA, et al. Human cytomegalovirus-encoded US9 targets MAVS and STING signaling to evade type I interferon immune responses. Nat Commun. 2018;9(1):125. 10.1038/s41467-017-02624-8. 29317664 10.1038/s41467-017-02624-8 PMC5760629

[90] Lee J-K, Kim J-E, Park BJ, Song Y-J. Human cytomegalovirus IE86 protein aa 136–289 mediates STING degradation and blocks the cGAS-STING pathway. J Microbiol. 2020;58:54–60. 10.1007/s12275-020-9577-6. 31898253 10.1007/s12275-020-9577-6

[91] Costa B, Becker J, Krammer T, Mulenge F, Durán V, Pavlou A, et al. Human cytomegalovirus exploits STING signaling and counteracts IFN/ISG induction to facilitate infection of dendritic cells. Nat Commun. 2024;15(1):1745. 10.1038/s41467-024-45614-3. 38409141 10.1038/s41467-024-45614-3 PMC10897438

[92] Fabits M, Gonçalves Magalhães V, Chan B, Girault V, Elbasani E, Rossetti E, et al. The cytomegalovirus tegument protein UL35 antagonizes pattern recognition receptor-mediated type I IFN transcription. Microorganisms. 2020;8(6): 790. 10.3390/microorganisms8060790. 32466380 10.3390/microorganisms8060790 PMC7356634

[93] Abate DA, Watanabe S, Mocarski ES. Major human cytomegalovirus structural protein pp65 (ppUL83) prevents interferon response factor 3 activation in the interferon response. J Virol. 2004;78(20):10995–1006. 10.1128/JVI.78.20.10995-11006.2004. 15452220 10.1128/JVI.78.20.10995-11006.2004 PMC521853

[94] Ren Y, Wang A, Zhang B, Ji W, Zhu X-X, Lou J, et al. Human cytomegalovirus UL36 inhibits IRF3-dependent immune signaling to counterbalance its immunoenhancement as apoptotic inhibitor. Sci Adv. 2023;9(40): eadi6586. 10.1126/sciadv.adi6586. 37792941 10.1126/sciadv.adi6586 PMC10550242

[95] Albright ER, Mickelson CK, Kalejta RF. Human cytomegalovirus ul138 protein inhibits the sting pathway and reduces interferon beta mrna accumulation during lytic and latent infections. Mbio. 2021;12(6):e02267-02221. 10.1128/mBio.02267-21. 34903048 10.1128/mBio.02267-21 PMC8669494

[96] Zhang Q, Song X, Ma P, Lv L, Zhang Y, Deng J, et al. Human cytomegalovirus miR-US33as-5p targets IFNAR1 to Achieve Immune Evasion during both lytic and latent infection. Front Immunol. 2021;12:12. 10.3389/fimmu.2021.628364. 10.3389/fimmu.2021.628364 PMC7973039 33746965

[97] Feng L, Sheng J, Vu GP, Liu Y, Foo C, Wu S, et al. Human cytomegalovirus UL23 inhibits transcription of interferon-γ stimulated genes and blocks antiviral interferon-γ responses by interacting with human N-myc interactor protein. PLoS Pathog. 2018;14(1): e1006867. 10.1371/journal.ppat.1006867. 29377960 10.1371/journal.ppat.1006867 PMC5805366

[98] Le-Trilling VTK, Becker T, Nachshon A, Stern-Ginossar N, Schöler L, Voigt S, et al. The human cytomegalovirus pUL145 isoforms Act as viral DDB1-Cullin-Associated factors to instruct host protein degradation to Impede Innate Immunity. Cell Rep. 2020;30(7):2248–60. 10.1016/j.celrep.2020.01.070. 32075763 10.1016/j.celrep.2020.01.070

[99] Kim YJ, Kim ET, Kim YE, Lee MK, Kwon KM, Kim KI, et al. Consecutive inhibition of ISG15 expression and ISGylation by Cytomegalovirus regulators. PLoS Pathog. 2016;12(8): e1005850. 10.1371/journal.ppat.1005850. 27564865 10.1371/journal.ppat.1005850 PMC5001722

[100] Goodwin CM, Schafer X, Munger J. UL26 attenuates IKKβ-mediated induction of interferon-stimulated gene (ISG) expression and enhanced protein ISGylation during human cytomegalovirus infection. J Virol. 2019;93(23):e01052–19. 10.1128/jvi. 31534044 10.1128/JVI.01052-19 PMC6854504

[101] Lee MK, Kim YJ, Kim Y-E, Han T-H, Milbradt J, Marschall M, et al. Transmembrane protein pUL50 of human cytomegalovirus inhibits ISGylation by downregulating UBE1L. J Virol. 2018;92(15):e00462-00418. 10.1128/jvi. 00462 – 18. 29743376 10.1128/JVI.00462-18 PMC6052311

[102] Cosman D, Müllberg J, Sutherland CL, Chin W, Armitage R, Fanslow W, et al. ULBPs, novel MHC class I–related molecules, bind to CMV glycoprotein UL16 and stimulate NK cytotoxicity through the NKG2D receptor. Immunity. 2001;14(2):123–33. 10.1016/s1074-7613(01)00095-4. 11239445 10.1016/s1074-7613(01)00095-4

[103] Chalupny NJ, Rein-Weston A, Dosch S, Cosman D. Down-regulation of the NKG2D ligand MICA by the human cytomegalovirus glycoprotein UL142. Biochem Biophys Res Commun. 2006;346(1):175–81. 10.1016/j.bbrc.2006.05.092. 16750166 10.1016/j.bbrc.2006.05.092

[104] Seidel E, Dassa L, Schuler C, Oiknine-Djian E, Wolf DG, Le-Trilling VTK, et al. The human cytomegalovirus protein UL147A downregulates the most prevalent MICA allele: MICA*008, to evade NK cell-mediated killing. PLoS Pathog. 2021;17(5):e1008807. 10.1371/journal.ppat.1008807. 33939764 10.1371/journal.ppat.1008807 PMC8118558

[105] Dassa L, Seidel E, Oiknine-Djian E, Yamin R, Wolf DG, Le-Trilling VTK, et al. The human cytomegalovirus protein UL148A downregulates the NK cell-activating ligand MICA to avoid NK Cell Attack. J Virol. 2018;92(17):e00162-00118. 10.1128/jvi.00162-18. 29950412 10.1128/JVI.00162-18 PMC6096798

[106] Nachmani D, Lankry D, Wolf DG, Mandelboim O. The human cytomegalovirus microRNA miR-UL112 acts synergistically with a cellular microRNA to escape immune elimination. Nat Immunol. 2010;11(9):806–13. 10.1038/ni.1916. 20694010 10.1038/ni.1916

[107] Tomasec P, Wang EC, Davison AJ, Vojtesek B, Armstrong M, Griffin C, et al. Downregulation of natural killer cell–activating ligand CD155 by human cytomegalovirus UL141. Nat Immunol. 2005;6(2):181–8. 10.1038/ni1156. 15640804 10.1038/ni1156 PMC2844263

[108] Kim Y, Park B, Cho S, Shin J, Cho K, Jun Y, et al. Human cytomegalovirus UL18 utilizes US6 for evading the NK and T-cell responses. PLoS Pathog. 2008;4(8): e1000123. 10.1371/journal.ppat.1000123. 18688275 10.1371/journal.ppat.1000123 PMC2483941

[109] Tomasec P, Braud VM, Rickards C, Powell MB, McSharry BP, Gadola S, et al. Surface expression of HLA-E, an inhibitor of natural killer cells, enhanced by human cytomegalovirus gpUL40. Science. 2000;287(5455):1031–3. 10.1126/science.287.5455.1031. 10669413 10.1126/science.287.5455.1031

[110] Yurochko AD. New mechanism by which human cytomegalovirus MicroRNAs negate the proinflammatory response to infection. mBio. 2017;8(2). 10.1128/mBio.00505-17. 10.1128/mBio.00505-17 PMC5395671 28420741

[111] Kim Y, Lee S, Kim S, Kim D, Ahn J-H, Ahn K. Human cytomegalovirus clinical strain-specific microRNA miR-UL148D targets the human chemokine RANTES during infection. PLoS Pathog. 2012;8(3): e1002577. 10.1371/journal.ppat.1002577. 22412377 10.1371/journal.ppat.1002577 PMC3297591

[112] Lüttichau HR. The cytomegalovirus UL146 gene product vCXCL1 targets both CXCR1 and CXCR2 as an agonist. J Biol Chem. 2010;285(12):9137–46. 10.1074/jbc.M109.002774. 20044480 10.1074/jbc.M109.002774 PMC2838333

[113] Skaletskaya A, Bartle LM, Chittenden T, McCormick AL, Mocarski ES, Goldmacher VS. A cytomegalovirus-encoded inhibitor of apoptosis that suppresses caspase-8 activation. Proceedings of the National Academy of Sciences. 2001;98(14):7829–34. 10.1073/pnas.141108798. 10.1073/pnas.141108798 PMC35427 11427719

[114] Goldmacher VS, Bartle LM, Skaletskaya A, Dionne CA, Kedersha NL, Vater CA, et al. A cytomegalovirus-encoded mitochondria-localized inhibitor of apoptosis structurally unrelated to Bcl-2. Proceedings of the National Academy of Sciences. 1999;96(22):12536–41. 10.1073/pnas.96.22.12536. 10.1073/pnas.96.22.12536 PMC22976 10535957

[115] Hildreth RL, Bullough MD, Zhang A, Chen H-L, Schwartz PH, Panchision DM, et al. Viral mitochondria-localized inhibitor of apoptosis (UL37 exon 1 protein) does not protect human neural precursor cells from human cytomegalovirus-induced cell death. J Gen Virol. 2012;93(11):2436–46. 10.1099/vir.0.044784-0. 22875256 10.1099/vir.0.044784-0 PMC4091285

[116] Kim HJ, Lee Y, Lee S, Park B. HCMV-encoded viral protein US12 promotes autophagy by inducing autophagy flux. Biochem Biophys Res Commun. 2023;654:94–101. 10.1016/j.bbrc.2023.03.004. 36898229 10.1016/j.bbrc.2023.03.004

[117] Tey SK, Khanna R. Autophagy mediates transporter associated with antigen processing-independent presentation of viral epitopes through MHC class I pathway. Blood J Am Soc Hematol. 2012;120(5):994–1004. 10.1182/blood-2012-01-402404. 10.1182/blood-2012-01-402404 22723550

[118] Mouna L, Hernandez E, Bonte D, Brost R, Amazit L, Delgui LR, et al. Analysis of the role of autophagy inhibition by two complementary human cytomegalovirus BECN1/Beclin 1-binding proteins. Autophagy. 2016;12(2):327–42. 10.1080/15548627.2015.1125071. 26654401 10.1080/15548627.2015.1125071 PMC4836022

[119] Wiertz EJ, Jones TR, Sun L, Bogyo M, Geuze HJ, Ploegh HL. The human cytomegalovirus US11 gene product dislocates MHC class I heavy chains from the endoplasmic reticulum to the cytosol. Cell. 1996;84(5):769–79. 10.1016/s0092-8674(00)81054-5. 8625414 10.1016/s0092-8674(00)81054-5

[120] Jones TR, Wiertz E, Sun L, Fish KN, Nelson JA, Ploegh HL. Human cytomegalovirus US3 impairs transport and maturation of major histocompatibility complex class I heavy chains. Proceedings of the National Academy of Sciences. 1996;93(21):11327–33. 10.1073/pnas.93.21.11327. 10.1073/pnas.93.21.11327 PMC38057 8876135

[121] Trgovcich J, Cebulla C, Zimmerman P, Sedmak DD. Human cytomegalovirus protein pp71 disrupts Major Histocompatibility Complex Class I Cell Surface expression. J Virol. 2006;80(2):951–63. 10.1128/jvi.80.2.951-963.2006. 16378997 10.1128/JVI.80.2.951-963.2006 PMC1346885

[122] Kim S, Lee S, Shin J, Kim Y, Evnouchidou I, Kim D, et al. Human cytomegalovirus microRNA miR-US4-1 inhibits CD8 + T cell responses by targeting the aminopeptidase ERAP1. Nat Immunol. 2011;12(10):984–91. 10.1038/ni.2097. 21892175 10.1038/ni.2097 PMC3526977

[123] Romania P, Cifaldi L, Pignoloni B, Starc N, D’Alicandro V, Melaiu O, et al. Identification of a genetic variation in ERAP1 aminopeptidase that prevents human cytomegalovirus miR-UL112-5p-mediated immunoevasion. Cell Rep. 2017;20(4):846–53. 10.1016/j.celrep.2017.06.084. 28746870 10.1016/j.celrep.2017.06.084

[124] Lau B, Poole E, Van Damme E, Bunkens L, Sowash M, King H, et al. Human cytomegalovirus miR-UL112-1 promotes the down-regulation of viral immediate early-gene expression during latency to prevent T-cell recognition of latently infected cells. J Gen Virol. 2016;97(9):2387–98. 10.1099/jgv.0.000546. 27411311 10.1099/jgv.0.000546 PMC5756489

[125] Hegde NR, Tomazin RA, Wisner TW, Dunn C, Boname JM, Lewinsohn DM, et al. Inhibition of HLA-DR assembly, transport, and loading by human cytomegalovirus glycoprotein US3: a novel mechanism for evading major histocompatibility complex class II antigen presentation. J Virol. 2002;76(21):10929–41. 10.1128/jvi.76.21.10929-10941.2002. 12368336 10.1128/JVI.76.21.10929-10941.2002 PMC136637

[126] Tomazin R, Boname J, Hegde NR, Lewinsohn DM, Altschuler Y, Jones TR, et al. Cytomegalovirus US2 destroys two components of the MHC class II pathway, preventing recognition by CD4 + T cells. Nat Med. 1999;5(9):1039–43. 10.1038/12478. 10470081 10.1038/12478

[127] Odeberg J, Plachter B, Brandén L, Söderberg-Nauclér C. Human cytomegalovirus protein pp65 mediates accumulation of HLA-DR in lysosomes and destruction of the HLA-DR α-chain. Blood. 2003;101(12):4870–7. 10.1182/blood-2002-05-1504. 12609847 10.1182/blood-2002-05-1504

[128] Spencer JV, Lockridge KM, Barry PA, Lin G, Tsang M, Penfold ME, et al. Potent immunosuppressive activities of cytomegalovirus-encoded interleukin-10. J Virol. 2002;76(3):1285–92. 10.1128/jvi.76.3.1285-1292.2002. 11773404 10.1128/JVI.76.3.1285-1292.2002 PMC135865

[129] Pleskoff O, Casarosa P, Verneuil L, Ainoun F, Beisser P, Smit M, et al. The human cytomegalovirus-encoded chemokine receptor US28 induces caspase‐dependent apoptosis. FEBS J. 2005;272(16):4163–77. 10.1111/j.1742-4658.2005.04829.x. 16098198 10.1111/j.1742-4658.2005.04829.x

[130] Berg C, Rosenkilde MM. Therapeutic targeting of HCMV-encoded chemokine receptor US28: Progress and challenges. Front Immunol. 2023;14:1135280. 10.3389/fimmu.2023.1135280. 36860859 10.3389/fimmu.2023.1135280 PMC9968965

[131] Yuan Q, Fan Z, Huang W, Huo X, Yang X, Ran Y, et al. Human cytomegalovirus UL23 exploits PD-L1 inhibitory signaling pathway to evade T cell-mediated cytotoxicity. Mbio. 2024;15:e01191-01124. 10.1128/mbio.01191-24. 38829126 10.1128/mbio.01191-24 PMC11253622

[132] Betsinger CN, Jankowski CSR, Hofstadter WA, Federspiel JD, Otter CJ, Jean Beltran PM, et al. The human cytomegalovirus protein pUL13 targets mitochondrial cristae architecture to increase cellular respiration during infection. Proc Natl Acad Sci U S A. 2021;118(32): e2101675118. 10.1073/pnas.2101675118. 34344827 10.1073/pnas.2101675118 PMC8364163

[133] Corrales-Aguilar E, Trilling M, Hunold K, Fiedler M, Le VTK, Reinhard H, et al. Human cytomegalovirus Fcγ binding proteins gp34 and gp68 antagonize Fcγ receptors I, II and III. PLoS Pathog. 2014;10(5): e1004131. 10.1371/journal.ppat.1004131. 24830376 10.1371/journal.ppat.1004131 PMC4022731

[134] Vezzani G, Pimazzoni S, Ferranti R, Calò S, Monda G, Amendola D, et al. Human immunoglobulins are transported to HCMV viral envelope by viral fc gamma receptors-dependent and independent mechanisms. Front Microbiol. 2023;13:13. 10.3389/fmicb.2022.1106401. 10.3389/fmicb.2022.1106401 PMC9885202 36726564

[135] Sprague ER, Reinhard H, Cheung EJ, Farley AH, Trujillo RD, Hengel H, et al. The human cytomegalovirus fc receptor gp68 binds the fc CH2-CH3 interface of immunoglobulin G. J Virol. 2008;82(7):3490–9. 10.1128/JVI.01476-07. 18216124 10.1128/JVI.01476-07 PMC2268481

[136] Berry R, Watson GM, Jonjic S, Degli-Esposti MA, Rossjohn J. Modulation of innate and adaptive immunity by cytomegaloviruses. Nat Rev Immunol. 2020;20(2):113–27. 10.1038/s41577-019-0225-5. 31666730 10.1038/s41577-019-0225-5

[137] Dauby N, Kummert C, Lecomte S, Liesnard C, Delforge M-L, Donner C, et al. Primary human cytomegalovirus infection induces the expansion of virus-specific activated and atypical memory B cells. J Infect Dis. 2014;210(8):1275–85. 10.1093/infdis/jiu255. 24795470 10.1093/infdis/jiu255

[138] Semmes EC, Miller IG, Rodgers N, Phan CT, Hurst JH, Walsh KM, et al. ADCC-activating antibodies correlate with decreased risk of congenital human cytomegalovirus transmission. JCI Insight. 2023;8(13). 10.1172/jci.insight.167768. 10.1172/jci.insight.167768 PMC10371338 37427588

[139] Chang WW, Barry PA, Szubin R, Wang D, Baumgarth N. Human cytomegalovirus suppresses type I interferon secretion by plasmacytoid dendritic cells through its interleukin 10 homolog. Virology. 2009;390(2):330–7. 10.1016/j.virol.2009.05.013. 19524994 10.1016/j.virol.2009.05.013 PMC2747589

[140] Avdic S, McSharry BP, Steain M, Poole E, Sinclair J, Abendroth A, et al. Human cytomegalovirus-encoded human interleukin-10 (IL-10) homolog amplifies its immunomodulatory potential by upregulating human IL-10 in monocytes. J Virol. 2016;90(8):3819–27. 10.1128/JVI.03066-15. 26792743 10.1128/JVI.03066-15 PMC4810557

[141] Moutaftsi M, Mehl AM, Borysiewicz LK, Tabi Z. Human cytomegalovirus inhibits maturation and impairs function of monocyte-derived dendritic cells. Blood J Am Soc Hematol. 2002;99(8):2913–21. 10.1182/blood.v99.8.2913. 10.1182/blood.v99.8.2913 11929782

[142] Moutaftsi M, Brennan P, Spector SA, Tabi Z. Impaired lymphoid chemokine-mediated migration due to a block on the chemokine receptor switch in human cytomegalovirus-infected dendritic cells. J Virol. 2004;78(6):3046–54. 10.1128/jvi.78.6.3046-3054.2004. 14990723 10.1128/JVI.78.6.3046-3054.2004 PMC353728

[143] Hancock MH, Hook LM, Mitchell J, Nelson JA. Human cytomegalovirus microRNAs miR-US5-1 and miR-UL112-3p block proinflammatory cytokine production in response to NF-κB-activating factors through direct downregulation of IKKα and IKKβ. mBio. 2017;8(2). 10.1128/mBio.00109-17. 10.1128/mBio.00109-17 PMC5340867 28270578

[144] Penfold ME, Dairaghi DJ, Duke GM, Saederup N, Mocarski ES, Kemble GW, et al. Cytomegalovirus encodes a potent α chemokine. Proceedings of the National Academy of Sciences. 1999;96(17):9839–44. 10.1073/pnas.96.17.9839. 10.1073/pnas.96.17.9839 PMC22297 10449781

[145] Hancock MH, Crawford LB, Pham AH, Mitchell J, Struthers HM, Yurochko AD, et al. Human cytomegalovirus miRNAs regulate TGF-β to mediate myelosuppression while maintaining viral latency in CD34 + hematopoietic progenitor cells. Cell Host Microbe. 2020;27(1):104–14. 10.1016/j.chom.2019.11.013. e4. 31866424 10.1016/j.chom.2019.11.013 PMC6952548

[146] Hancock MH, Mitchell J, Goodrum FD, Nelson JA. Human cytomegalovirus miR-US5-2 downregulation of GAB1 regulates cellular proliferation and UL138 expression through modulation of epidermal growth factor receptor signaling pathways. Msphere. 2020;5(4):e00582-00520. 10.1128/mSphere.00582-20. 32759334 10.1128/mSphere.00582-20 PMC7407068

[147] Mikell I, Crawford LB, Hancock MH, Mitchell J, Buehler J, Goodrum F, et al. HCMV miR-US22 down-regulation of EGR-1 regulates CD34 + hematopoietic progenitor cell proliferation and viral reactivation. PLoS Pathog. 2019;15(11): e1007854. 10.1371/journal.ppat.1007854. 31725809 10.1371/journal.ppat.1007854 PMC6855405

[148] Reeves M, Lehner P, Sissons J, Sinclair J. An in vitro model for the regulation of human cytomegalovirus latency and reactivation in dendritic cells by chromatin remodelling. J Gen Virol. 2005;86(11):2949–54. 10.1099/vir.0.81161-0. 16227215 10.1099/vir.0.81161-0

[149] Wang Z, Wang G, Lu H, Li H, Tang M, Tong A. Development of therapeutic antibodies for the treatment of diseases. Mol Biomed. 2022;3(1):35. 10.1186/s43556-022-00100-4. 36418786 10.1186/s43556-022-00100-4 PMC9684400

[150] Talavera-Barber M, Flint K, Graber B, Dhital R, Kaptsan I, Medoro AK, et al. Antibody titers against human cytomegalovirus gM/gN and gB among pregnant women and their infants. Front Pead. 2022;10. 10.3389/fped.2022.846254. 10.3389/fped.2022.846254 PMC9259787 35813379

[151] Pötzsch S, Spindler N, Wiegers A-K, Fisch T, Rücker P, Sticht H, et al. B cell repertoire analysis identifies new antigenic domains on glycoprotein B of human cytomegalovirus which are target of neutralizing antibodies. PLoS Pathog. 2011;7(8): e1002172. 10.1371/journal.ppat.1002172. 21852946 10.1371/journal.ppat.1002172 PMC3154849

[152] Spindler N, Diestel U, Stump JD, Wiegers A-K, Winkler TH, Sticht H, et al. Structural basis for the Recognition of Human Cytomegalovirus glycoprotein B by a neutralizing human antibody. PLoS Pathog. 2014;10(10): e1004377. 10.1371/journal.ppat.1004377. 25299639 10.1371/journal.ppat.1004377 PMC4192593

[153] Su H, Ye X, Freed DC, Li L, Ku Z, Xiong W, et al. Potent bispecific neutralizing antibody targeting glycoprotein B and the gH/gL/pUL128/130/131 complex of human cytomegalovirus. Antimicrob Agents Chemother. 2021;65(3):e02422-02420. 10.1128/AAC.02422-20. 33361306 10.1128/AAC.02422-20 PMC8092496

[154] McVoy MM, Tenorio E, Kauvar LM. A native human monoclonal antibody targeting HCMV gB (AD-2 site I). Int J Mol Sci. 2018;19(12):3982. 10.3390/ijms19123982. 30544903 10.3390/ijms19123982 PMC6321246

[155] Ye X, Ku Z, Zhang N, Fu T-M, An Z. Recent progress in development of monoclonal antibodies against human cytomegalovirus. Curr Opin Virol. 2022;52:166–73. 10.1016/j.coviro.2021.12.002. 34952264 10.1016/j.coviro.2021.12.002

[156] Sponholtz MR, Byrne PO, Lee AG, Ramamohan AR, Goldsmith JA, McCool RS, et al. Structure-based design of a soluble human cytomegalovirus glycoprotein B antigen stabilized in a prefusion-like conformation. Proceedings of the National Academy of Sciences. 2024;121(37):e2404250121. 10.1073/pnas.2404250121. 10.1073/pnas.2404250121 PMC11406251 39231203

[157] Gerna G, Percivalle E, Perez L, Lanzavecchia A, Lilleri D. Monoclonal antibodies to different components of the human cytomegalovirus (HCMV) Pentamer gH/gL/pUL128L and trimer gH/gL/gO as well as Antibodies Elicited during primary HCMV infection prevent epithelial cell syncytium formation. J Virol. 2016;90(14):6216–23. 10.1128/JVI.00121-16. 27122579 10.1128/JVI.00121-16 PMC4936130

[158] Fouts AE, Comps-Agrar L, Stengel KF, Ellerman D, Schoeffler AJ, Warming S, et al. Mechanism for neutralizing activity by the anti-CMV gH/gL monoclonal antibody MSL-109. Proc Natl Acad Sci U S A. 2014;111(22):8209–14. 10.1073/pnas.1404653111. 24843144 10.1073/pnas.1404653111 PMC4050585

[159] Ai Y, Wu C, Zhang M, Jaijyan DK, Liu T, Zan L, et al. Neutralization epitopes in Trimer and Pentamer complexes recognized by potent cytomegalovirus-neutralizing human monoclonal antibodies. Microbiol Spectr. 2022;10: e0139322. 10.1128/spectrum.01393-22. 36342276 10.1128/spectrum.01393-22 PMC9784774

[160] Zehner M, Alt M, Ashurov A, Goldsmith JA, Spies R, Weiler N, et al. Single-cell analysis of memory B cells from top neutralizers reveals multiple sites of vulnerability within HCMV Trimer and Pentamer. Immunity. 2023;56(11):2602-e2010. 10.1016/j.immuni.2023.10.009. 37967532 10.1016/j.immuni.2023.10.009

[161] Ha S, Li F, Troutman MC, Freed DC, Tang A, Loughney JW, et al. Neutralization of Diverse Human cytomegalovirus strains conferred by antibodies targeting viral gH/gL/pUL128-131 Pentameric Complex. J Virol. 2017;91(7): e02033-16. 10.1128/JVI.02033-16. 28077654 10.1128/JVI.02033-16 PMC5355600

[162] Macagno A, Bernasconi NL, Vanzetta F, Dander E, Sarasini A, Revello MG, et al. Isolation of human monoclonal antibodies that potently neutralize human cytomegalovirus infection by targeting different epitopes on the gH/gL/UL128-131A complex. J Virol. 2010;84(2):1005–13. 10.1128/JVI.01809-09. 19889756 10.1128/JVI.01809-09 PMC2798344

[163] Boeckh M, Bowden RA, Storer B, Chao NJ, Spielberger R, Tierney DK, et al. Randomized, placebo-controlled, double-blind study of a cytomegalovirus-specific monoclonal antibody (MSL-109) for prevention of cytomegalovirus infection after allogeneic hematopoietic stem cell transplantation. Biol Blood Marrow Transplant. 2001;7(6):343–51. 10.1016/S1083-8791(01)80005-7. 11464977 10.1016/s1083-8791(01)80005-7

[164] Xia L, Tang A, Meng W, Freed DC, He L, Wang D, et al. Active evolution of memory B-cells specific to viral gH/gL/pUL128/130/131 pentameric complex in healthy subjects with silent human cytomegalovirus infection. Oncotarget. 2017;8(43). 10.18632/oncotarget.18359. 10.18632/oncotarget.18359 PMC5650289 29088734

[165] Kauvar LM, Liu K, Park M, DeChene N, Stephenson R, Tenorio E, et al. A High-Affinity native human antibody neutralizes human cytomegalovirus infection of diverse cell types. Antimicrob Agents Chemother. 2015;59(3):1558–68. 10.1128/AAC.04295-14. 25534746 10.1128/AAC.04295-14 PMC4325823

[166] Ohlin M, Sundqvist VA, Mach M, Wahren B, Borrebaeck CA. Fine specificity of the human immune response to the major neutralization epitopes expressed on cytomegalovirus gp58/116 (gB), as determined with human monoclonal antibodies. J Virol. 1993;67(2):703–10. 10.1128/JVI.67.2.703-710.1993. 7678304 10.1128/jvi.67.2.703-710.1993 PMC237421

[167] Tian Y, Hu D, Li Y, Yang L. Development of therapeutic vaccines for the treatment of diseases. Mol Biomed. 2022;3(1):40. 10.1186/s43556-022-00098-9. 36477638 10.1186/s43556-022-00098-9 PMC9729511

[168] Das R, Blázquez-Gamero D, Bernstein DI, Gantt S, Bautista O, Beck K, et al. Safety, efficacy, and immunogenicity of a replication-defective human cytomegalovirus vaccine, V160, in cytomegalovirus-seronegative women: a double-blind, randomised, placebo-controlled, phase 2b trial. Lancet Infect Dis. 2023;23(12):1383–94. 10.1016/S1473-3099(23)00343-2. 37660711 10.1016/S1473-3099(23)00343-2

[169] Gomes AC, Baraniak IA, Lankina A, Moulder Z, Holenya P, Atkinson C, et al. The cytomegalovirus gB/MF59 vaccine candidate induces antibodies against an antigenic domain controlling cell-to-cell spread. Nat Commun. 2023;14(1):1041. 10.1038/s41467-023-36683-x. 36823200 10.1038/s41467-023-36683-x PMC9950427

[170] Sponholtz M, Byrne P, Lee A, Ramamohan A, Goldsmith J, McCool R, et al. Structure-based design of a soluble human cytomegalovirus glycoprotein B antigen stabilized in a prefusion-like conformation. 2024. 10.1101/2024.02.10.579772. 10.1073/pnas.2404250121 PMC11406251 39231203

[171] Jacobson MA, Adler SP, Sinclair E, Black D, Smith A, Chu A, et al. A CMV DNA vaccine primes for memory immune responses to live-attenuated CMV (Towne strain). Vaccine. 2009;27(10):1540–8. 10.1016/j.vaccine.2009.01.006. 19168107 10.1016/j.vaccine.2009.01.006

[172] Wloch MK, Smith LR, Boutsaboualoy S, Reyes L, Han C, Kehler J, et al. Safety and immunogenicity of a bivalent cytomegalovirus DNA vaccine in healthy adult subjects. J Infect Dis. 2008;197(12):1634–42. 10.1086/588385. 18444883 10.1086/588385 PMC2956065

[173] Kharfan-Dabaja MA, Boeckh M, Wilck MB, Langston AA, Chu AH, Wloch MK, et al. A novel therapeutic cytomegalovirus DNA vaccine in allogeneic haemopoietic stem-cell transplantation: a randomised, double-blind, placebo-controlled, phase 2 trial. Lancet Infect Dis. 2012;12(4):290–9. 10.1016/s1473-3099(11)70344-9. 22237175 10.1016/S1473-3099(11)70344-9

[174] Ljungman P, Bermudez A, Logan AC, Kharfan-Dabaja MA, Chevallier P, Martino R, et al. A randomised, placebo-controlled phase 3 study to evaluate the efficacy and safety of ASP0113, a DNA-based CMV vaccine, in seropositive allogeneic haematopoietic cell transplant recipients. eClinicalMedicine. 2021;33. 10.1016/j.eclinm.2021.100787. 10.1016/j.eclinm.2021.100787 PMC8020145 33842870

[175] Wu K, Hou YJ, Makrinos D, Liu R, Zhu A, Koch M, et al. Characterization of humoral and cellular immunologic responses to an mRNA-based human cytomegalovirus vaccine from a phase 1 trial of healthy adults. J Virol. 2024;98(4):e01603-01623. 10.1128/jvi.01603-23. 38526054 10.1128/jvi.01603-23 PMC11019844

[176] Hu X, Karthigeyan KP, Herbek S, Valencia SM, Jenks JA, Webster H, et al. Human cytomegalovirus mRNA-1647 vaccine candidate elicits potent and broad neutralization and higher antibody-dependent Cellular cytotoxicity responses than the gB/MF59 vaccine. J Infect Dis. 2024. 10.1093/infdis/jiad593. 38324766 10.1093/infdis/jiad593 PMC11326847

[177] Langley JM, Gantt S, Halperin SA, Ward B, McNeil S, Ye L, et al. An enveloped virus-like particle alum-adjuvanted cytomegalovirus vaccine is safe and immunogenic: a first-in-humans Canadian Immunization Research Network (CIRN) study. Vaccine. 2024;42(3):713–22. 10.1016/j.vaccine.2023.12.019. 38142214 10.1016/j.vaccine.2023.12.019

[178] Schwendinger M, Thiry G, De Vos B, Leroux-Roels G, Bruhwyler J, Huygens A, et al. A randomized dose-escalating phase I trial of a replication-deficient lymphocytic choriomeningitis virus vector-based vaccine against human cytomegalovirus. J Infect Dis. 2020;225(8):1399–410. 10.1093/infdis/jiaa121. 10.1093/infdis/jiaa121 PMC9016443 32313928

[179] Bernstein DI, Reap EA, Katen K, Watson A, Smith K, Norberg P, et al. Randomized, double-blind, phase 1 trial of an alphavirus replicon vaccine for cytomegalovirus in CMV seronegative adult volunteers. Vaccine. 2009;28(2):484–93. 10.1016/j.vaccine.2009.09.135. 19857446 10.1016/j.vaccine.2009.09.135

[180] Gourin C, Alain S, Hantz S. Anti-CMV therapy, what next? A systematic review. Front Microbiol. 2023;14: 1321116. 10.3389/fmicb.2023.1321116. 38053548 10.3389/fmicb.2023.1321116 PMC10694278

[181] Fishman JA. Infection in solid-organ transplant recipients. N Engl J Med. 2007;357(25):2601–14. 10.1056/NEJMra064928. 18094380 10.1056/NEJMra064928

[182] Mercorelli B, Sinigalia E, Loregian A, Palù G. Human cytomegalovirus DNA replication: antiviral targets and drugs. Rev Med Virol. 2008;18(3):177–210. 10.1002/rmv.558. 18027349 10.1002/rmv.558

[183] Hayes M, Boge CL, Sharova A, Vader D, Mitrou M, Galetaki DM, et al. Antiviral toxicities in pediatric solid organ transplant recipients. Am J Transplant. 2022;22(12):3012–20. 10.1111/ajt.17171. 35971847 10.1111/ajt.17171

[184] Clercq ED, Holý A. Acyclic nucleoside phosphonates: a key class of antiviral drugs. Nat Rev Drug Discovery. 2005;4(11):928–40. 10.1038/nrd1877. 16264436 10.1038/nrd1877

[185] Ljungman P, Griffiths P, Paya C. Definitions of cytomegalovirus infection and disease in transplant recipients. Clin Infect Dis. 2002;34(8):1094–7. 10.1093/cid/ciw668. 11914998 10.1086/339329

[186] Stern A, Alonso CD, Garcia-Vidal C, Cardozo C, Slavin M, Yong MK, et al. Safety and efficacy of intravenously administered cidofovir in adult haematopoietic cell transplant recipients: a retrospective multicentre cohort study. J Antimicrob Chemother. 2021;76(11):3020–8. 10.1093/jac/dkab259. 34324678 10.1093/jac/dkab259 PMC8677452

[187] Painter W, Robertson A, Trost LC, Godkin S, Lampert B, Painter G. First Pharmacokinetic and Safety Study in humans of the Novel lipid antiviral conjugate CMX001, a broad-spectrum oral drug active against double-stranded DNA viruses. Antimicrob Agents Chemother. 2012;56(5):2726–34. 10.1128/aac.05983-11. 22391537 10.1128/AAC.05983-11 PMC3346600

[188] Marty FM, Winston DJ, Chemaly RF, Mullane KM, Shore TB, Papanicolaou GA, et al. A randomized, double-blind, placebo-controlled phase 3 trial of oral brincidofovir for cytomegalovirus prophylaxis in allogeneic hematopoietic cell transplantation. Biol Blood Marrow Transplant. 2019;25(2):369–81. 10.1016/j.bbmt.2018.09.038. 30292744 10.1016/j.bbmt.2018.09.038 PMC8196624

[189] Kern ER, Kushner NL, Hartline CB, Williams-Aziz SL, Harden EA, Zhou S, et al. In vitro activity and mechanism of action of methylenecyclopropane analogs of nucleosides against herpesvirus replication. Antimicrob Agents Chemother. 2005;49(3):1039–45. 10.1128/AAC.49.3.1039-1045.2005. 15728900 10.1128/AAC.49.3.1039-1045.2005 PMC549243

[190] James SH, Hartline CB, Harden EA, Driebe EM, Schupp JM, Engelthaler DM, et al. Cyclopropavir inhibits the normal function of the human cytomegalovirus UL97 kinase. Antimicrob Agents Chemother. 2011;55(10):4682–91. 10.1128/AAC.00571-11. 21788463 10.1128/AAC.00571-11 PMC3186952

[191] Wagstaff AJ, Bryson HM. Foscarnet: a reappraisal of its antiviral activity, pharmacokinetic properties and therapeutic use in immunocompromised patients with viral infections. Drugs. 1994;48:199–226. 10.2165/00003495-199448020-00007. 7527325 10.2165/00003495-199448020-00007

[192] Crumpacker CS, Ganciclovir. N Engl J Med. 1996;335(10):721–9. 10.1056/NEJM199609053351007. 8786764 10.1056/NEJM199609053351007

[193] Goldner T, Hewlett G, Ettischer N, Ruebsamen-Schaeff H, Zimmermann H, Lischka P. The novel anticytomegalovirus compound AIC246 (Letermovir) inhibits human cytomegalovirus replication through a specific antiviral mechanism that involves the viral terminase. J Virol. 2011;85(20):10884–93. 10.1128/JVI.05265-11. 21752907 10.1128/JVI.05265-11 PMC3187482

[194] Marty FM, Ljungman P, Chemaly RF, Maertens J, Dadwal SS, Duarte RF, et al. Letermovir prophylaxis for cytomegalovirus in hematopoietic-cell transplantation. N Engl J Med. 2017;377(25):2433–44. 10.1056/NEJMoa1706640. 29211658 10.1056/NEJMoa1706640

[195] Underwood MR, Harvey RJ, Stanat SC, Hemphill ML, Miller T, Drach JC, et al. Inhibition of human cytomegalovirus DNA maturation by a Benzimidazole Ribonucleoside is mediated through the UL89 gene product. J Virol. 1998;72(1):717–25. 10.1128/jvi.72.1.717-725.1998. 9420278 10.1128/jvi.72.1.717-725.1998 PMC109427

[196] Evers DL, Komazin G, Shin D, Hwang DD, Townsend LB, Drach JC. Interactions among antiviral drugs acting late in the replication cycle of human cytomegalovirus. Antiviral Res. 2002;56(1):61–72. 10.1016/S0166-3542(02)00094-3. 12323400 10.1016/s0166-3542(02)00094-3

[197] Reefschlaeger J, Bender W, Hallenberger S, Weber O, Eckenberg P, Goldmann S, et al. Novel non-nucleoside inhibitors of cytomegaloviruses (BAY 38-4766): in vitro and in vivo antiviral activity and mechanism of action. J Antimicrob Chemother. 2001;48(6):757–67. 10.1093/jac/48.6.757. 11733458 10.1093/jac/48.6.757

[198] Biron KK, Harvey RJ, Chamberlain SC, Good SS, Smith AA, Davis MG, et al. Potent and selective inhibition of human cytomegalovirus replication by 1263w94, a benzimidazole l-riboside with a unique mode of action. Antimicrob Agents Chemother. 2002;46(8):2365–72. 10.1128/aac.46.8.2365-2372.2002. 12121906 10.1128/AAC.46.8.2365-2372.2002 PMC127361

[199] Chou S, Marousek GI. Maribavir antagonizes the antiviral action of ganciclovir on human cytomegalovirus. Antimicrob Agents Chemother. 2006;50(10):3470–2. 10.1128/aac.00577-06. 17005835 10.1128/AAC.00577-06 PMC1610080

[200] Kang C, Maribavir. First Approval Drugs. 2022;82(3):335–40. 10.1007/s40265-022-01677-4. 35147913 10.1007/s40265-022-01677-4

[201] Avery RK, Alain S, Alexander BD, Blumberg EA, Chemaly RF, Cordonnier C, et al. Maribavir for refractory cytomegalovirus infections with or without Resistance Post-transplant: results from a phase 3 Randomized Clinical Trial. Clin Infect Dis. 2021;75(4):690–701. 10.1093/cid/ciab988. 10.1093/cid/ciab988 PMC9464078 34864943

[202] Huang Y, Yang Y, Liu G, Xu M. New clinical application prospects of artemisinin and its derivatives: a scoping review. Infect Dis Poverty. 2023;12(1):115. 10.1186/s40249-023-01152-6. 38072951 10.1186/s40249-023-01152-6 PMC10712159

[203] Hutterer C, Niemann I, Milbradt J, Fröhlich T, Reiter C, Kadioglu O, et al. The broad-spectrum antiinfective drug artesunate interferes with the canonical nuclear factor kappa B (NF-κB) pathway by targeting RelA/p65. Antiviral Res. 2015;124:101–9. 10.1016/j.antiviral.2015.10.003. 26546752 10.1016/j.antiviral.2015.10.003

[204] Germi R, Mariette C, Alain S, Lupo J, Thiebaut A, Brion JP, et al. Success and failure of artesunate treatment in five transplant recipients with disease caused by drug-resistant cytomegalovirus. Antiviral Res. 2014;101:57–61. 10.1016/j.antiviral.2013.10.014. 24184983 10.1016/j.antiviral.2013.10.014

[205] Chou S, Marousek G, Auerochs S, Stamminger T, Milbradt J, Marschall M. The unique antiviral activity of artesunate is broadly effective against human cytomegaloviruses including therapy-resistant mutants. Antiviral Res. 2011;92(2):364–8. 10.1016/j.antiviral.2011.07.018. 21843554 10.1016/j.antiviral.2011.07.018

[206] Cotin S, Calliste C-A, Mazeron M-C, Hantz S, Duroux J-L, Rawlinson WD, et al. Eight flavonoids and their potential as inhibitors of human cytomegalovirus replication. Antiviral Res. 2012;96(2):181–6. 10.1016/j.antiviral.2012.09.010. 23000494 10.1016/j.antiviral.2012.09.010

[207] Andouard D, Gueye R, Hantz S, Fagnère C, Liagre B, Bernardaud L, et al. Impact of new cyclooxygenase 2 inhibitors on human cytomegalovirus replication in vitro. Antivir Ther. 2021;26(6–8):117–25. 10.1177/13596535211064078. 35485337 10.1177/13596535211064078

[208] Zhu H, Cong JP, Yu D, Bresnahan WA, Shenk TE. Inhibition of cyclooxygenase 2 blocks human cytomegalovirus replication. Proceedings of the National Academy of Sciences. 2002;99(6):3932–7. 10.1073/pnas.052713799. 10.1073/pnas.052713799 PMC122626 11867761

[209] Yi HA, Kim MS, Jang SY, Lee YM, Ahn JH, Lee CH. Cellular signals involved in cyclooxygenase-2 expression induced by human cytomegalovirus. Virus Res. 2009;146(1):89–96. 10.1016/j.virusres.2009.09.004. 19748535 10.1016/j.virusres.2009.09.004

[210] Waldman WJ, Knight DA, Blinder L, Shen J, Lurain NS, Miller DM, et al. Inhibition of Cytomegalovirus in vitro and in vivo by the experimental immunosuppressive Agent Leflunomide. Intervirology. 2000;42(5–6):412–8. 10.1159/000053979. 10.1159/000053979 10702725

[211] John GT, Manivannan J, Chandy S, Peter S, Fleming DH, Chandy SJ, et al. A prospective evaluation of leflunomide therapy for cytomegalovirus disease in renal transplant recipients. Transplantation Proceedings. 2005;37(10):4303–5. 10.1016/j.transproceed.2005.10.116. 16387103 10.1016/j.transproceed.2005.10.116

[212] Tedesco-Silva H, Pascual J, Viklicky O, Basic-Jukic N, Cassuto E, Kim DY, et al. Safety of everolimus with reduced calcineurin inhibitor exposure in De Novo kidney transplants: an analysis from the Randomized TRANSFORM Study. Transplantation. 2019;103(9):1953–63. 10.1097/tp.0000000000002626. 30801548 10.1097/TP.0000000000002626

[213] Roy S, Arav-Boger R. New cell-signaling pathways for controlling cytomegalovirus replication. Am J Transplant. 2014;14(6):1249–58. 10.1111/ajt.12725. 24839861 10.1111/ajt.12725 PMC4280670

[214] Ma C, Chen P, Du J, Wang L, Lu N, Sun J, et al. Adoptive transfer of CMV-specific TCR-T cells for the treatment of CMV infection after haploidentical hematopoietic stem cell transplantation. J Immunother Cancer. 2024;12(1): e007735. 10.1136/jitc-2023-007735. 38184303 10.1136/jitc-2023-007735 PMC10773422

[215] Chao M, Daihong L, Dou L. Adoptive transfer of CMV-Specific TCR-T cells for the treatment of CMV infection after Haploidentical hematopoietic stem cell transplantation. Blood. 2023;142:2117. 10.1182/blood-2023-184405. 10.1136/jitc-2023-007735 PMC10773422 38184303

[216] Liu G, Chen H, Cao X, Jia L, Rui W, Zheng H, et al. Efficacy of pp65-specific TCR-T cell therapy in treating cytomegalovirus infection after hematopoietic stem cell transplantation. Am J Hematol. 2022;97(11):1453–63. 10.1002/ajh.26708. 36054234 10.1002/ajh.26708

[217] Long X, Zhang Z, Li Y, Deng K, Gao W, Huang M, et al. ScRNA-seq reveals novel immune-suppressive T cells and investigates CMV-TCR-T cells cytotoxicity against GBM. J Immunother Cancer. 2024;12(4): e008967. 10.1136/jitc-2024-008967. 38688579 10.1136/jitc-2024-008967 PMC11086384

[218] Shen L, Yang J, Zuo C, Xu J, Ma L, He Q, et al. Circular mRNA-based TCR-T offers a safe and effective therapeutic strategy for treatment of cytomegalovirus infection. Mol Ther. 2024;32(1):168–84. 10.1016/j.ymthe.2023.11.017. 37974400 10.1016/j.ymthe.2023.11.017 PMC10787193

